# Molecular detection of *Rickettsia* spp. in ticks (Ixodida: Argasidae and Ixodidae) and mites (Trombiculoidae) from a Brazilian taxonomic collection

**DOI:** 10.1007/s10493-026-01200-4

**Published:** 2026-10-28

**Authors:** Almir R. Pepato, Nikolay V. Anisimov, Jefferson C. Carvalho Farias da Silva, Teofania H. D. Amorim Vidigal, Marcelo B. Labruna, Brenda K. Gomes-Almeida

**Affiliations:** 1 Pós-Graduação Em Zoologia/Laboratório de Sistemática E Evolução de Ácaros Acariformes, Departamento de Zoologia, Instituto de Ciências Biológicas, Universidade Federal de Minas Gerais (UFMG). Av. Antônio Carlos, Pampulha, Belo Horizonte, Minas Gerais 6627 Brazil; 2 Department of Biochemistry, Institute of Pharmacy, Tyumen State Medical University, Tyumen, Russia; 3 Institute X-Bio, Tyumen State University, Tyumen, Russia; 4 Departamento de Medicina Veterinária Preventiva E Saúde Animal, Faculdade de Medicina Veterinária E Zootecnia da, Universidade de São Paulo-FMVZ-USP, São Paulo, SP Brazil

**Keywords:** Taxonomic collections, One Health, Ectoparasites, Vertebrates, Taxonomy, Cave, Bat, gltA

## Abstract

*Rickettsia* species are arthropod-hosted endosymbionts classified into phylogenetic and serological groups, heterogeneous concerning their ecologies. This study aims to screen ticks and mites deposited in the Acarological Collection at Center of Taxonomical Collections at Universidade Federal de Minas Gerais, Brazil, for rickettsial occurrences, making available genetic data on hosts and bacteria. Endogenous mitochondrial markers for ticks and mitochondrial and nuclear genes for chiggers were sequenced for sample quality control and species identification. A total of 38 soft ticks belonging to the genus *Ornithodoros* (at least six putative species), 18 hard ticks belonging to the genus *Amblyomma* (7 spp)*,* and 52 chiggers belonging to genus *Whartonia* (2 spp) led to sequences. Polymerase Chain reactions for a Citrate Synthase gene fragment led to 21 sequences grouped in the phylogroups Bellii, Spotted Fever, and Transitional, with one sequence of *Rickettsia* obtained from *Amblyomma dubitatum* (identified as *R. bellii*), eight sequences from *Ornithodoros* (classified in six gltA genotypes associated to the phylogroups Bellii, Spotted Fever and Transitional) and twelve associate to *Whartonia pachywhartoni* (three likely in the Spotted Fever and nine in the Transitional phylogroup). Considering the diversity of rickettsial occurrences in *Ornithodoros*, with four of the nine gltA genotypes detected only in *O. fonsecai*, a species known to bite humans, the medical relevance of *Rickettsia* associated with caves and bats needs to be further investigated.

**Supplementary Information:**

The online version contains supplementary material available at https://doi.org/10.1007/s10493-026-01200-4.

## Introduction

The Alphaproteobacteria genus *Rickettsia* belongs to the diversified order Rickettsiales, which includes many intracellular obligate symbionts (Dumler and Walker 2005), a condition apparently convergently acquired (Castelli et al. 2024). The evolution of the ability to invade and hijack eukaryotic cells along the trend toward losing pathways for aminoacid and nucleotide synthesis make them unable to survive extracellularly, turning a group difficult to laboratory manipulation and the use of traditional techniques for bacterial study (McGinn and Lamason 2021).

On other hand, since some species of *Rickettsia* cause life-threatening diseases such as the Rocky Mountain spotted fever and typhus, the genus has attracted considerable attention, particularly in medicine (Raoult and Roux 1997; Parola et al. 2005), prompting molecular studies on their infection mechanisms (Uchiyama et al. 2006; Ge and Rikihisa 2011).The genus *Rickettsia* encompasses a broad diversity of hosts and infection mechanisms, extending beyond its potential threat to human and other vertebrates (Perlman et al. 2006; Weinert et al. 2009; McGinn and Lamason 2021), with the transmission to humans and animals, mainly through arthropod bites such as mites, ticks and insect hosts, with a predominance in ticks (Parola et al. 2013; El Karkouri et al. 2022). Moreover, their close relationship to mitochondria makes them key models in evolutionary biology (Fitzpatrick et al. 2006; El Karkouri et al. 2022).

Overall, using multiloci and phylogenomic analysis, the genera could be divided in a few major phylogroups: Spotted Fever group (SFG), the Transitional group (TRG) the Typhus group (TG), the Canadensis group (CG), and the Bellii group (BG) as well some less diversified lineages such as Scapularis, Adalia, Helvetica, Meloidiae and Rhyzobius. These lineages were recovered in multiloci and genomic scale phylogenetic analyses in reasonably congruent topologies, which branching order will be used to order an overview on the genus diversity below (Murray et al. 2016; Perlman et al. 2006; Weinert et al. 2009; El Karkouri et al. 2022; Davison et al. 2022).

Former groups Torix and Megaira are recently reputed as separate genera (Davison et al. 2022). They have the largest range of hosts, from microeukaryotes to arthropods. Remaining *Rickettsia* are arthropod symbionts, and comprise the arthropod borne pathogens. Infections of basal *Rickettsia* led to a diversity of phenotypes including male-killing (Hurst and Jiggins 2000), parthenogenesis induction (Stouthamer et al. 2001), mutualists required for oogenesis (Perotti et al. 2006), facultative mutualism (Jaenike 2012), and other mechanisms of sex-ratio distortion (Takahashi et al. 1997; Weinert et al. 2007; Himler et al. 2011).

The basal groups Rhyzobius and Meloidae are both named after beetles, even though each also comprise Dipteran as hosts. Despite fragmentary knowledge on both lineages, populations of *Rhyzobius litura* have female-biased sex-ratios, suggesting sex-ratio distortion (Weinert et al. 2007).

The following branch from *Rickettsia* tree is the Bellii group, all associated with arthropods, comprising hexapoda (Diptera, Coleoptera, Hemiptera, Hymenoptera, Lepidoptera and Neuroptera), ticks (Ixodidae and Argasidae), and mites (Mesostigmata). The Bellii group is associated with a wide range of adaptive phenotypes, including parthenogenesis induction, male-killing, uncharacterized sex-ratio distortion, requirement for oogenesis and facultative mutualism, and possible vertebrate pathogenicity (Weinert 2015). This group includes the single *Rickettsia* that acts as a plant pathogen with potential horizontal transmission through a plant host (Davis et al. 1998).

The Adalia group is named after ladybird beetles but also includes Dipteran and Collembola hosts (Weinert et al. 2009), the occurrence of male-killing species in the Adalia and Bellii groups is probably homoplastic (Weinert 2015). The Canadensis group, includes bacteria with hard tick hosts (Ixodidae), and the beetle *Coccotrypes dactyliperda*, which is sterile when cured of *Rickettsia* and *Wolbachia* (Zchori-Fein et al. 2006), but if the *Rickettsia* is responsible for this phenomena, it will be homoplastic with representatives of the Torix and Transitional groups (Weinert 2015).

The following groups have their internal relationships with low support and incongruence among datasets, despite being included in a clade with high support (Murray et al. 2016; Perlman et al. 2006; Weinert et al. 2009; El Karkouri et al. 2022; Davison et al. 2022).

Historically and in current clinical practice, the SFG and TG groups were recognized based on serological characteristics (Stewart and Stewart 2021).

The SFG includes, among others, *R. montanensis*, *R. massiliae*, *R. japonica*, *R. peacockii*, *R. rickettsii*, *R. conorii*, *R. sibirica*, and *R. parkeri* (Weinert et al. 2009; Davison et al. 2022). All members are associated with ticks (Ixodida), and includes the first recognized and one of the most lethal pathogens among *Rickettsia*, *R. rickettsii* (Parola et al. 2013). On other hand, the pathogenicity of *R. parkeri* was not recognized until 60 years after its description, being responsible for milder cases of Spotted fever (Parola et al. 2005). The group includes an even more interesting case, *R. peacockii*. The species is perpetuated transstadially and transovarially in ticks, and the presence of *R. peacockii* within ovaries interfering in the ability of *R. rickettsii* to infect the ovarian tissues and to be transovarially transmitted to progeny (Parola et al. 2005). However, *R. peacockii* does not invade the salivary glands, precluding its transmission during biting (Niebylski et al. 1997), and possesses an *ompA* gene that contains three premature stop codons, hence is unable to produce a functional *ompA* protein, crucial for pathogenicity (Baldridge et al. 2004).

The Typhus group comprises two named species, *Rickettsia typhi* and *Rickettsia prowazekii,* primarily associated with fleas (Siphonaptera) and lice (Phthiraptera) (Parola et al. 2005).

The Transitional group contains named species such as *R. felis*, *R. australis*, and *R. akari*, and lineages occurring associated to Ixodida, Diptera, Hemiptera, Hymenoptera, Psocoptera, and Siphonaptera (Weinert 2015). *Rickettsia felis* is vectored by cat fleas to cats and humans, to which it has been associated with fevers and spots, while a closely related strain is required for oogenesis in the booklouse and is thought to cause parthenogenesis (Thepparit et al. 2011). *Rickettsia australis* causes Queensland tick typhus, transmitted to humans through the bites of the hard ticks *Ixodes holocyclus* or *Ixodes tasmani*. It has mostly mild symptoms – fever, headache, and myalgia followed by the development of a maculopapular or vesicular rash, an inoculation eschar (65% of cases), and lymphadenopathy (71%) (McBride et al. 2007). Finally, the closely related *R. akari*, the causative agent of rickettsialpox, has as invertebrate host mites belonging to the order Mesostigmata, specifically *Liponyssoides sanguineus*, despite it is suspected of being transmitted by ticks too (e.g. Iweriebor et al. 2017).

Mites belonging to the epifamily Trombiculoidae (= Trombiculidae sensu Kudryashova 1998) include species with larvae parasite of vertebrates, with a few exceptions (with invertebrate hosts) that are likely reversals (Costa et al. 2024; Felska et al. 2018; Vasconcelos et al. 2017). They differ from other ectoparasite mites for feeding upon host tissues through the stylostome, a straw-like structure produced by interaction of mite saliva and host tissue (Shatrov 2009). Furthermore harboring *Orientia tsutsugamushi* and other members of genus *Orientia* (Martínez-Valdebenito et al. 2024), bacteria belonging to the genus *Rickettsia* are frequent in Trombiculoidae mites, being detected in chiggers feeding on small mammals and birds in Brazil, USA, Slovakia, and in many places across Asia (Choi et al. 2007; Tsui et al. 2007; Huang et al. 2017; Miťková et al. 2015; Jacinavicius et al. 2019; Linsuwanon et al. 2021; Kuo et al. 2022; Ponnusamy et al. 2022; Bassini-Silva et al. 2018; 2023). Most of them were grouped in the Transitional group, implying that these bacteria in Trombiculoidae may be arthropod symbionts, vertebrate pathogens or both. Some molecularly detected lineages, such as ‘*Rickettsia* sp. clone MB74-1’ and ‘*Rickettsia* sp. TwKM02’ and related bacteria occurring in South Korea seems to be specific to chiggers and are seldom found in the rodent host or coinfesting ectoparasites (Choi et al. 2007; Kuo et al. 2022; Tsui et al. 2007).

### Acarological taxonomic collections as source of genetic information on hosts and pathogens

Natural History collections are an untapped source of biodiversity information, including the occurrence, ecological and genetic data, even on populations extinct by antropic action (Kapun et al 2025). The specimens currently in natural history collections differ widely concerning their potential in granting access to the genetic data.

Raxworthy and Smith (2021) name all DNA stored in collections as “Archival DNA”, and distinguish among them Historical DNA (hDNA) and Modern DNA. The former is the DNA fortuitously preserved in the tissues of traditional voucher specimens accumulated since natural history collections inception (mostly <200 years old), while the later are samples designed to preserve as much as possible DNA for sequencing, including keeping individuals or tissue samples in alcohol or buffers, refrigerated and protected from light (mostly collected in the last 40 years).

In addition to the collection target group, there are many examples of successful access to the genetic material of pathogens (e.g. Nelder et al. 2024) or microbiota (e.g. Nambiar et al. 2025) associated to natural history collection specimens. According to Stärk and colleagues (2015): “One Health surveillance describes the systematic collection, validation, analysis, interpretation of data and dissemination of information collected on humans, animals and the environment to inform decisions for more effective, evidence- and system-based health interventions”. It is clear from its very definition that many aspects of this approach may interplay with the objectives of taxonomic collections and the access to their genetic information content, which harbors relevant samples from natural or synanthropic communities, including potential vectors and hosts of symbionts.

This study aims to access the “Archival DNA” of an acarological collection including samples that were not originally intended to downstream molecular studies, and screening them for *Rickettsia* in a “One Healthy” perspective. Despite this study relies exclusively on specimens available in a museum collection rather than on a systematic sampling strategy, which constrains the ability to draw robust ecological or epidemiological inferences about *Rickettsia* diversity and distribution, it creates the foundation upon which future studies can be planned, identifying priority areas for sampling and establishing expectations regarding the diversity of hosts and bacteria.

## Material and methods

### Specimen sampling and collection

The Acarological collection at “Centro de Coleções Taxonômicas da UFMG” – Acronym UFMG-AC (Zhang 2018) – is a repository started in 2012, totaling, to October/2023, 16,361 specimens, mounted on slides or in alcohol (Pepato 2024). These mites were collected in all regions of Brazil and several countries in the Americas, Europe, Asia and Oceania. The collection stands out for its sampling of mites from caves and coastal habitats, comprising approximately 150 different families, providing a solid basis for taxonomic and ecological studies, and type material (Pepato 2024). The cave dwelling mites include a substantial sample of members of Epifamily Trombiculoidae and Order Ixodida, mainly from Minas Gerais State.

As the original sampling context of most mites was not that of employing molecular techniques, but an inventory of invertebrate communities, they were initially preserved for days to months in 70% ethanol at room temperature before having the fixative replaced by absolute alcohol and kept at − 20 °C upon arrival at UFMG-AC.

### DNA extraction and morphological identification

Genomic DNA was extracted from single specimens using QIAamp® DNA Micro kit (Qiagen) for larvae and the Wizard® Genomic DNA Purification Kit (Promega) for nymph and adult stages, following the manufacturer’s protocol, except for using two steps of the final elution in the former, leading to a final volume of 50 μl, and by removing and preserving the specimen’s exoskeleton after the protein digestion step.

Most exoskeletons recovered after DNA extraction were mounted on permanent microscope slides using Hoyer’s medium, following Krantz and Walter (2009), and kept in an oven at 50–55 °C for approximately one week or until completely dry. Slides were examined under a Leica DM2500 light microscope equipped with an ICC50 W digital camera and phase contrast optics. In the case of post larval ticks, specimens were preserved in 70% ethanol and carefully examined under Leica M125 stereomicroscopes, as described by Muñoz-Leal et al. (2017).

Ticks and mites were identified using morphological keys and original species descriptions, for post larval stages of hard ticks (Ixodidae) following Barros-Battesti et al. (2006) and Martins et al. (2010) and soft tick larvae and post larval (Argasidae) by Dantas-Torres et al. (2019). Since the generic classification of Argasidae remains under debate (Mans et al. 2021; Kneubehl et al. 2022), in the present study we adopted the classification of Guglielmone et al. (2003) sensu Hoogstraal (1985) for the Argasidae genera, until this taxonomic problem is solved.

Gomes-Almeida et al. (2023) included most of the chiggers reported in this study and was consulted for the identification of two species of the genus *Whartonia*.

### PCR amplification of host genes and Rickettsia screening, sequencing and chromatogram checking

Primer sequences and cycling conditions were summarized in Table 1.

**Table 1:** Primers and thermal cycles used in the polymerase chain reactions (PCR) employed in this study. D_0_, Initial denaturation; D, Denaturation; A, Annealing; E, Extension; E_f_, Final extension. For nested PCR (COI), 2nd step primers had M13 forward (TGT AAA ACG ACG GCC AGT) and reverse (CAG GAA ACA GCTA TGA CC) tails added to employ them for sequencing

	Primer pair	Program	
16SrDNA Mitocondrial (Ticks) ~410 bp (Mangold et al. 1998)			
Single step PCR	16S+1: CTG CTC AAT GAT TTT TTA AAT TGC TGT GG; 16S-1: CCG GTC TGA ACT CAG ATC AAG T	D_0_ 94 °C, 2’; 7x (D 94 °C, 30″; A 47 °C+0.3 °C, 35″; E 72 °C, 45″); 28x (D 94 °C, 30″; A 50 °C, 35″; E 72 °C, 45″); E_f_ 72 °C, 5’	
COI Mitochondrial (Ticks and Mites) ~1200 bp (Klimov et al. 2018; Klimov and O’Connor 2009)			
Nested 1st step	COX1 16F: TGA NTW TTT TCH ACW AAY CAY AA; COX1 1324R: CDG WRT AHC GDC GDG GTA T	D_0_ 94 °C, 2’; 10x (D 94 °C, 30″; A 40 °C, 1’; E 72 °C, 1′40″); 25x (D 94 °C, 30″; A 48 °C, 35″; E 72 °C, 2’); E_f_ 72 °C, 7’	
Nested 2nd step	COX1 25Fshort: CHA CWA AYC AYA ARR AYA; COX1 1282R: CCW VYT ARD CCT ARR AAR TGT TG	D_0_ 94 °C, 2’; 20x (D 94 °C, 30″; A 49 °C, 35″; E 72 °C, 2’); 18x (D 94 °C, 30″; A 52 °C, 35″; E 72 °C, 2’); E_f_ 72 °C, 7’	
COI Mitochondrial (Mites) ~1000 bp (Pepato et al. 2019)			
Nested 1st step	COX1 220F: ATA ATH GGD GGD TTY GGI AA; COX1 1324R	D_0_ 94 °C, 2’; 38x (D 94 °C, 30″; A 45 °C, 60″; E 72 °C, 90″); E_f_ 72 °C, 5’	
Nested 2nd step	COX1 270F: TGA YAT RGC NTW YCC ICG; COX1 1282R	D_0_ 94 °C, 2’; 10x (D 94 °C, 30″; A 48 °C, 35″; E 72 °C, 2’); 18x (D 94 °C, 30″; A 51 °C, 35″; E 72 °C, 2’); E_f_ 72 °C, 7’	
COI Mitochondrial (Mites) ~ 600 bp (Costa et al. 2019)			
Single step PCR	COI Forw: GAT TTT TTG GTC ACC CAG AAG; COI 2R: GGR TAR TCW GAR TAW GGN CGW GGT AT	D_0_ 94 °C, 2’; 10x (D 94 °C, 30″; A 47+0.3 °C, 35″; E 72 °C, 1’); 28x (D 94 °C, 30″; A 50 °C, 35″; E 72 °C, 1’); E_f_ 72 °C, 5’	
28S D3-D5 ~860 (Klimov et al. 2018)			
Nested 1st step	28S f1 4F: GAC CTC AGAT CAR GCG AGA H 28S P1 3R GCT GTT CAC ATG RAA CCC TTC	D_0_ 94 °C, 2’; 35x (D 94 °C, 30″; A 50 °C, 30″; E 72 °C, 2′15″+1″); E_f_ 72 °C, 5’	
Nested 2nd step	28Sa DD: GAC CCG TCT TGA AAC ACG GA 28SIR acar2: TGC TAC TAC CAC CAA GAT CTG C	D_0_ 94 °C, 2’; 35x (D 94 °C, 30″; A 53 °C, 25″; E 72 °C, 80″); E_f_ 72 °C, 5’	
28S D3-D4 (Mites) ~620 bp (Gomes-Almeida et al. 2023)			
Single step PCR	28Sa DD; R1446: GTT GTT ACA CAC TCC TTA GCG G	D_0_ 94 °C, 2’; 38x (D 94 °C, 30″; A 50 °C, 30″; E 72 °C, 60″); E_f_ 72 °C, 5’	
28 s D3 (Mites) ~300 bp (Whiting et al. 1997)			
Single step PCR	28Sa DD; 28S B: TCG GAA GGA ACC AGC TAC TA	D_0_ 94 °C, 2’; 38x (D 94 °C, 30″; A 50 °C, 30″; E 72 °C, 40″); E_f_ 72 °C, 5’	
gltA Citrate synthase (*Rickettsia* spp) ~350 bp (Labruna et al. 2004)			
Single step PCR	CS78: GCA AGT ATC GGT GAG GAT GTA AT; CS323: GCT TCC TTA AAA TTC AAT AAA TCA GGA T	D_0_ 94 °C, 2’; 40x (D 94 °C, 15″; A 48 °C, 30″; E 72 °C, 30″); E_f_ 72 °C, 7’	
16SrDNA (*Rickettsia* spp) ~ 930 bp (Weinert et al. 2009; Lane 1991)			
Single step PCR	27f: AGA GTT TGA TCC TGG CTC AG; rssur: GAA AGC ATC TCT GCG ATC CG	D_0_ 94 °C, 3’; 34x (D 94 °C, 30″; A 60 °C, 30″; E 72 °C, 45″); E_f_ 72 °C, 7’	
ATP synthase F1 alpha subunit (*Rickettsia* spp) ~ 440 bp (Weinert et al. 2009; Vitorino et al. 2007)			
Single step PCR	atpAf2: ATC AAG CGT TGC ACA GAT AG; Vitr: CGA CTT ACC GAA ATA CCG AC	D_0_ 94 °C, 3’; 35x (D 94 °C, 30″; A 56 °C, 30″; E 72 °C, 1’); E_f_ 72 °C, 7’	
ATP synthase F1 alpha subunit (*Rickettsia* spp) ~ 300 bp (Weinert et al. 2009)			
Single step PCR	atpAf2; atpA536r: GGA AGT GCC GTA AGT GAA CC	D_0_ 94 °C, 3’; 35x (D 94 °C, 30″; A 58 °C, 30″; E 72 °C, 1’); E_f_ 72 °C, 7’	
coxA subunit I of cytochrome C oxidase (*Rickettsia* spp) ~ 800 bp (Weinert et al. 2009)			
Single step PCR	CoxAf2: ACA GCC GTT GAT ATG GCT A; coxA1413r: CAT ATT CCA ACC GGC AAA AG	D_0_ 94 °C, 3’; 35x (D 94 °C, 30″; A 58 °C, 30″; E 72 °C, 1’); E_f_ 72 °C, 7’	
coxA subunit I of cytochrome C oxidase (*Rickettsia* spp) ~ 1000 bp (Weinert et al. 2009)			
Single step PCR	CoxA322f: GGT GCT CCT GAT ATG GCA TT; CoxAr1: CAT ATT CCA GCC GGC AAA AG	D_0_ 94 °C, 3’; 35x (D 94 °C, 30″; A 58 °C, 30″; E 72 °C, 1’); E_f_ 72 °C, 7’	

For host DNA quality checking and barcoding, amplification of fragments of the mitochondrial large ribosomal subunit (16S) for ticks, mitochondrial cytochrome c oxidase subunit I (COI) for mites and ticks, and nuclear large ribosomal subunit (28S) for mites were attempted.

Samples sequenced for host genes were screened for *Rickettsia* spp. using conventional PCR targeting a fragment of the citrate synthase (*gltA*) gene. Extractions from which *gltA* sequences could be obtained were used in attempts to obtain additional loci, in order to further characterize the lineages detected. Using the same primers as Weinert et al. (2009), we attempted to obtain sequences from the rickettsial genes 16SrDNA (Small Subunit Ribosomal DNA), *atpA* (ATP synthase subunit alpha), and *coxA* (cytochrome C oxidase subunit I) to include detected lineages in multiloci phylogenetic analyses.

All PCR reactions were performed in 20 μl of final volume with Platinum Taq DNA Polymerase (Invitrogen) in a Mastercycler nexus (Eppendorf) thermocycler. The master mix for initial PCR contained 2.0 μl of PCR buffer (1X), 1.4 μl MgCl 2 (50 mM), 1.4 μL of dNTP (10 mM each) and 0.8 μl of each oligonucleotide primer (10uM), to which 1–3 μl of genomic DNA or alternatively 0.5 μl of parent PCR products for nested reactions was added. All reactions included a negative control (ultrapure water) and, for *Rickettsi*a spp. screening, a positive control (*Rickettsia rickettsii*).

All PCR products found positive in 1% agarose gel electrophoresis were purified using the Ampure® (Agencourt) kit and sequenced using a 3730 DNA Analyzer and BigDyeTM Terminator v3.1 (Applied Biosystems) according to the manufacturer’s protocol.

Forward and reverse chromatograms were checked, edited and assembled into contigs using software ChromasPro 1.41 (Technelysium Pty Ltd). All sequences generated for this study were compared with available mites or bacteria sequences using the NCBI BLAST feature (https://blast.ncbi.nlm.nih.gov/Blast.cgi) (Altschul et al. 1997) and deposited in the GenBank database. All new sequences are summarized in the supplementary Tables S1, S2, and S4.

### Alignment and phylogenetic inference for host identification

The mitochondrial 16S and COI sequences were employed in an integrative approach to assist tick identification. Mitochondrial 16S rDNA were aligned in MAFFT (Katoh et al. 2002) using the E-INS-i strategy (Katoh et al. 2002) implemented in the MAFFT server at https://mafft.cbrc.jp/alignment/server/, employing the 200 PAM/K=2 scoring matrix for nucleotide sequences. Sequences from soft ticks were combined to those from all Argasidae species recorded from Brazil according to Dantas-Torres et al. (2019), and an undescribed putative new species reported in Jorge et al. (2022), referred by the authors as *Ornithodoros* sp. Ubajara. Hard ticks sequences were combined to sequences deposited in the GenBank from species identified morphologically in this study.

Mitochondrial COI sequences were aligned in Clustal and translated into aminoacids using MEGA X (Kumar et al. 2018) just to trim low quality extremities and check for stop codons. Hard and soft ticks had their COI sequences combined in a single alignment that also included sequences retrieved from GenBank based on previous morphological identification and NCBI BLAST searches (Altschul et al. 1997).

Finally, for Trombiculoidae mitochondrial COI and nuclear 28S rDNA were employed for DNA extraction quality check. Most samples were already included in Gomes-Almeida et al. (2023), comprising the species *Whartonia pachywhartoni* Vercammen-Grandjean, 1966 and *W. nudosetosa* (Wharton, 1938). Sequences are summarized in Supplementary Table S2.

Alignment features and distances were calculated using MEGA X (Kumar et al. 2018). The model for sequence evolution was chosen using the Bayesian Information Criterion, calculated in ModelFinder (Kalyaanamoorthy et al. 2017) implemented in IQ-TREE 3 (Wong et al. 2025). IQ-TREE 3 also was employed for inferring the maximum likelihood tree, and Ultrafast Bootstrap and the SHlike approximate likelihood ratio test (SH-aLRT), both calculated with 1000 replicates. Mitochondrial 16S rDNA tree was employed as input for PTP Maximum Likelihood species delimitation analyses (Zhang et al. 2013), at the bPTP webserver (https://species.h-its.org/ptp/).

#### *Screening for *Rickettsia* and phylogenetic analyses of bacterial markers*

The sequences from *gltA* gene were combined with sequences from GenBank to identify the affinities of the *Rickettsia* detected, as summarized in Supplementary Table S3: (A) representatives of the main lineages of *Rickettsia*, including basal groups Rhyzobius and Meloidae; (B) Sequences from *Rickettsia* detected in chiggers, as reviewed by Chaisiri et al 2023; (C) Matches with sequences deposited in GenBank using the online implemented BLATn, during the preliminary sequence processing described above. After preliminary automatic alignment with the aid of the BioEdit 7.2.1 software (Hall 1999) sequences had their extremities trimmed and checked for the maintenance of the reading frame in the program MEGA X (Kumar et al. 2018).

Multiloci alignment included those terminals for which at least two markers could be obtained and built on Weinert et al. (2009) study. GenBank accessions for retrieved and newly obtained sequences are summarized in Supplementary Table S4. Sequences of coding genes were aligned using Clustal, checked for stop codons and trimmed in BioEdit 7.2.1 software (Hall 1999), except rickettsial 16S rDNA that, despite being a non-coding region, presented a straightforward alignment. Markers were concatenated using FASconCAT-G program (Kück and Meusemann 2010).

The best fit model was chosen according the Bayesian Information Criterion, calculated in ModelFinder (Kalyaanamoorthy et al. 2017) implemented in IQ-TREE 3 (Wong et al. 2025). While the single marker *gltA* gene analyses were treated as a single partition, each gene in the multiloci analyses was set in a different partition and the bestfit partitioning scheme was chosen by merging them (command -m MFP+MERGE). These models were employed in Bayesian and maximum likelihood phylogenetic performed in MrBayes 3.2.7 (Ronquist et al. 2012) and in IQ-TREE 3 (Wong et al. 2025), respectively. Each Bayesian analysis was run in two independent and simultaneous runs with four chains each, comprising 10^7^ generations sampled every 1000th for gltA data, and 2×10^8^ generation sampled each 500 generations for multiloci data, with the first 12% sampled generations discarded as burnin in both cases. Convergence was evaluated by the Average Standard Deviation of Split Frequencies < 0.01, and a Expected Sample Size > 200 checked in Tracer 1.7 (Rambaut et al. 2018). Screen shots from this program were included among the supplementary material.

## Results

**Fig. 1:**
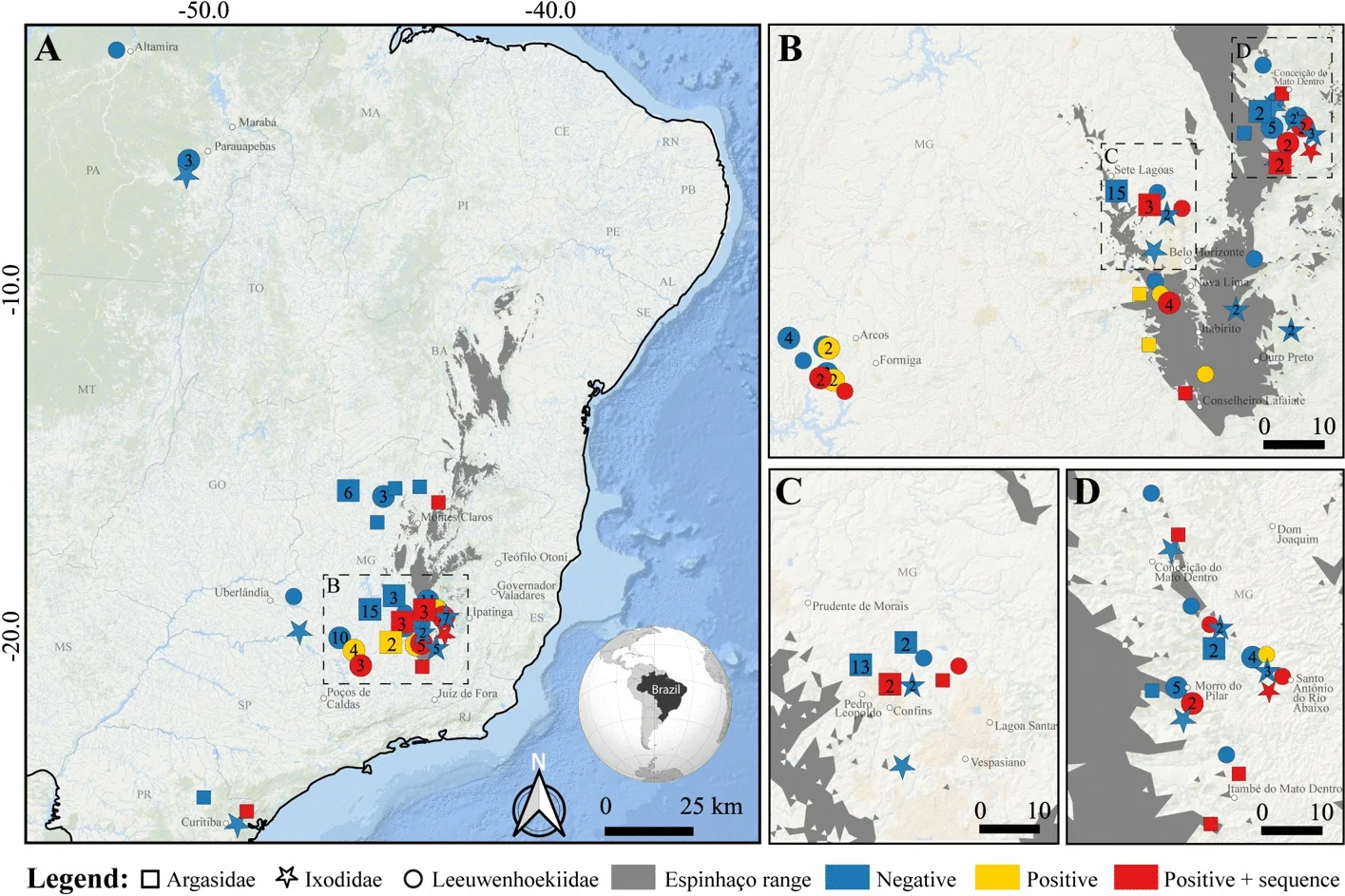
Map depicting sampling localities for tested mites with negative results for *Rickettsia* (blue), positive in electrophoresis gel but that failed sequencing (yellow) and successfully sequenced individuals (red). **A**– All sampling sites across Brazil; **B**– Minas Gerais state sites, highlighting overlapping sampling points; **C**– Detail of southern Espinhaço Range (Quadrilátero Ferrífero region); **D**– Detail of central Espinhaço Range. Squares: Argasidae; stars: Ixodidae; and circles: Trombiculoidae. Espinhaço Range is shaded in gray

Detection attempts were performed in a total of 363 specimens, comprising 158 ticks (Ixodida: Argasidae and Ixodidae) and 205 mites (Trombiculoidae), including larvae, nymphs and adults (List of collection numbers in the Supplementary material). These specimens were collected in localities in the Brazilian states of Minas Gerais, Pará, and São Paulo, most of them (327) from caves or cave associated fauna. Among them, only those that led to sequences were depicted in Fig. 1, and listed in Supplementary Tables S1 and S2. A total of 38 soft ticks belonging to the genus *Ornithodoros*, 18 hard ticks belonging to the genus *Amblyomma,* and 52 chiggers belonging to genus *Whartonia* led to sequences.

### Arthropod hosts identification

**Fig. 2:**
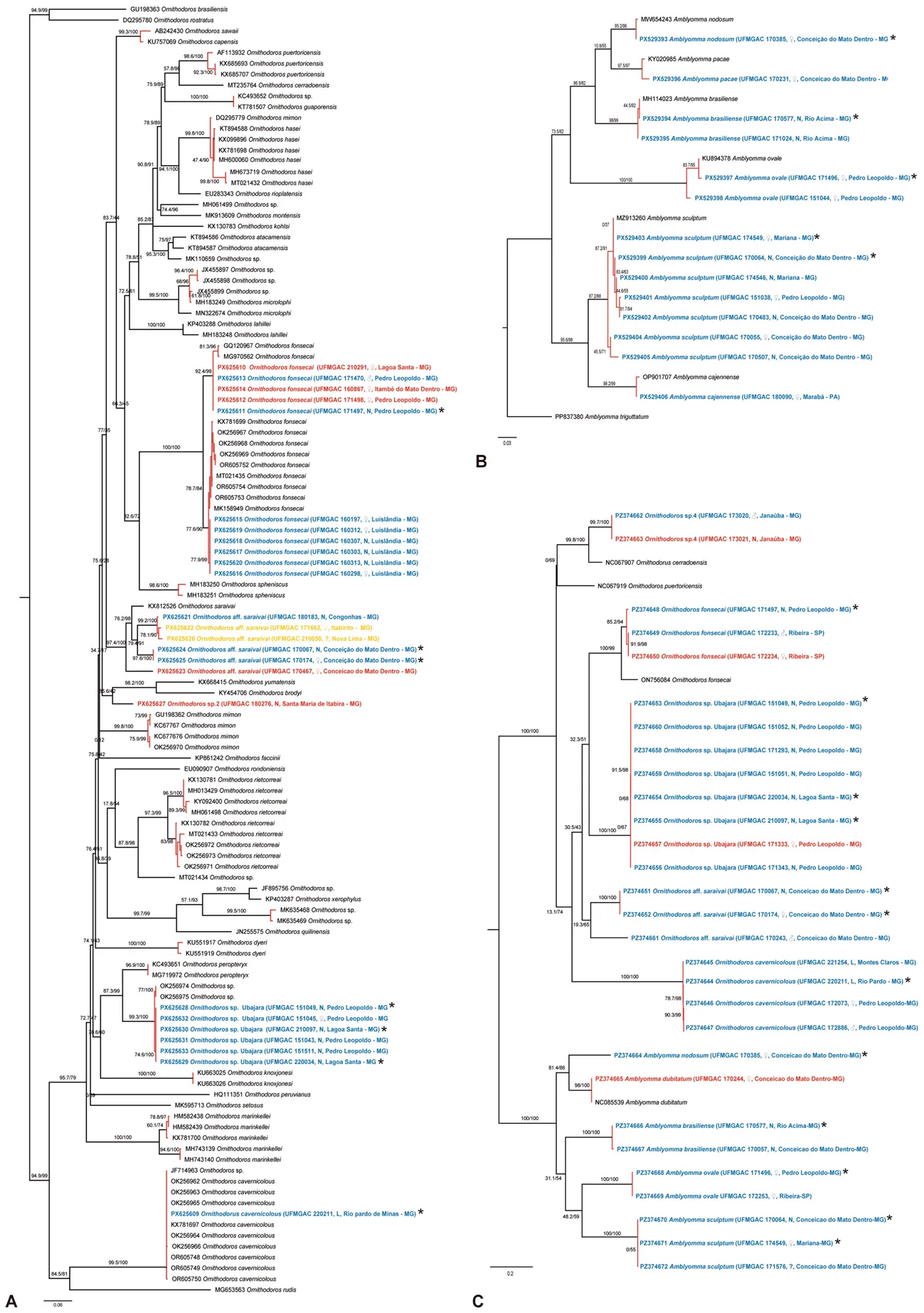
**A**- Maximum likelihood phylogenetic tree including mitochondrial 16S rDNA sequences from Brazilian *Ornithodoros* (Argasidae) species along newly obtained sequences; **B-** Maximum likelihood phylogenetic tree including mitochondrial 16S rDNA sequences from *Amblyomma* (Ixodidae) newly obtained sequences along co-specific sequences available in GenBank; **C**- Maximum likelihood phylogenetic tree including mitochondrial COI sequences of all newly sequenced Argasidae and Ixodidae, plus the sequences available in GenBank with close hits in BLASTn. Values associated with branches are SHlike approximate likelihood ratio test (SH-aLRT) and UltraFast Bootstrap (UFBoot), respectively, both calculated with 1000 replicates. The newly sequenced terminals are indicated in blue for those negative for rickettsial *gltA*, yellow for positive amplification but failed sequencing, and red for *Rickettsia* successfully sequenced. *, indicates terminals sequenced both to 16S and COI

Sequences of mitochondrial 16S and COI were employed for molecular species delimitation in ticks (Fig. 2). Concerning Argasidade, all belonging to the genus *Ornithodoros*, a total of 25 specimens were sequenced for 16S, 20 for COI, and seven were sequenced for both markers. The aligned 16S new sequences are 424–418 bps long, counting 135 variable positions out 432 positions, among them 99 parsimony informative. The COI nucleotide sequences counted 330 variable positions out of 973 bps, among them 320 parsimony informative. Translated into aminoacids, it led to 43 out 324 positions, 39 parsimony informative.

Among Argasidae, the species *O. cavernicolous* Dantas-Torres, Venzal & Labruna, 2012 and *O. fonsecai* (Labruna and Venzal) could be identified both morphologically and molecularly beyond any reasonable doubt. The presence of cheeks and legs with micromammillate cuticle points these ticks as members of the mostly bat-associated soft ticks of the subgenus *Alectorobiu*s (Dantas-Torres et al. 2012; Labruna and Venzal 2009). Both species were originally described from specimens obtained in caves or associated with bats, exactly as in our case. Similar to Oliveira et al (2024), 16S sequences from *O. fonsecai* grouped into two haplogroups, those obtained from Itambé do Mato Dentro, Lagoa Santa and Pedro Leopoldo Municipalities grouped with sequences from Mato Grosso do Sul state – Cerrado biome, type locality – and Rio de Janeiro – Atlantic Forest biome, whereas the specimens from the Caatinga – Pernambuco and Ceará states – and Cerrado biomes – Mato Grosso state – grouped with sequences of ticks from Luislândia municipality (Fig. 2A). Despite all *O. fonsecai* sequenced came from the same state, the later municipality is in a dry region, distant ~ 560 kms from the other localities, hence the division between an haplogroup inhabiting drier environments and other to more humidity seems to stand. The highest p-distance observed among terminals from the two haplogroups is 0.043, observed between the sequence from Mato Grosso do Sul (GQ120967) and sequences from Ceará state (KX781699, OK256967). Among sequences within both haplogroups was observed an average p-distance = 0.006.

Molecular data also clearly grouped ticks collected in caves from Minas Gerais with the undescribed “*Ornithodoros* sp. Ubajara”, referred this way after the Ubajara municipality, Ceará State, Northeast Brazil, where it was obtained from caves in the semi arid Caatinga biome (Jorge et al. 2022). Their 16S sequences have an average distance p = 0.005, and PTP analysis clearly recovers them as co-specific.

Six 16S sequences grouped with *Ornithodoros saraivai* Muñoz-Leal & Labruna, 2017 (Fig. 1A), but neither PTP nor the pairwise distances p= 0.079–0.050, support unambiguously assigning the specimens to such species. A least 16S sequence from an individual was recovered as sister to clade including *Ornithodoros yumatensis* Cooley & Kohls, 1941 (GenBank: KX668415) and *Ornithodoros brodyi* Matheson, 1935 (GenBank: KY454706), but with even higher pairwise distances (pairwise p = 0.121 and p = 0.138), suggestive of a new species. These ticks should be further investigated taxonomically.

Most individuals sequenced for COI could be unambiguously assigned to *O. cavernicolous*, *O. fonsecai*, and *Ornithodoros* sp. Ubajara thanks terminals sequenced for both 16S and COI (Fig. 2C). In contrast to the 16S marker, there was no available sequence of the COI marker for the type specimens of *O. saraivai*. Even so, of the specimens morphologically identified as *O. saraivai* and confirmed by 16S sequences (Fig. 2A), at least three of them, all from Conceição do Mato Dentro, generated COI sequences that formed a distinct clade (Fig. 2C), in which one of the sequences showed a considerable p-distance (= 0.136) and without PTP support for being cospecific. Two ticks from Janaúba municipality, Minas Gerais state, morphologically identified as *Ornithodoros* sp., was recovered in a clade along *Ornithodoros cerradoensis* Muñoz-Leal, Martins & Labruna, 2020 (NC_067907, average p-distance = 0.102).

All ixodid belonging to *Amblyomma* could be identified to the species level, both through molecules and morphology, comprising seven species: *A. brasiliense* Aragão, 1908 (N=3), *A. cajennense* (Fabricius, 1787) (N=1), *A. dubitatum* Neumann, 1899 (N=1), *A. nodosum* Neumann, 1899 (N=1), *A. ovale* Kock, 1844 (N=3), *A. pacae* Aragão, 1911 (N=1), and *A. sculptum* Berlese, 1888 (N= 8).

The same for *Whartonia*, with most host sequences already reported in previous studies, with 44 individuals identified as *W. pachywhartoni* Vercammen-Grandjean, 1966 and eight as *W. nudosetosa* (Wharton, 1938).

### *Rickettsia* detection

A total of 36 samples tested positive for *Rickettsia* spp. from ticks (Ixodida: *Amblyomma dubitatum* and Argasidae: *Ornithodoros* spp.) and mites (Trombiculoidae: *Whartonia pachywhartoni*), all collected from cave soil or bats (Fig. 1). From these samples, 21 *gltA* gene partial sequences were successfully obtained (Supplementary Table S1-S2).

The *gltA* alignment, after adding GenBank terminals (Supplementary Table S3) comprised 77 sequences, 379 columns, 165 variable, and 125 parsimony-informative sites. The best-fit model was the K3Pu+F+G4 (Kimura 3-parameter model with unequal base frequencies and a gamma distribution) chosen according to BIC. In MrBayes analyses, it was approximate to the slightly more parameterized GTR+F+G4.

**Fig. 3:**
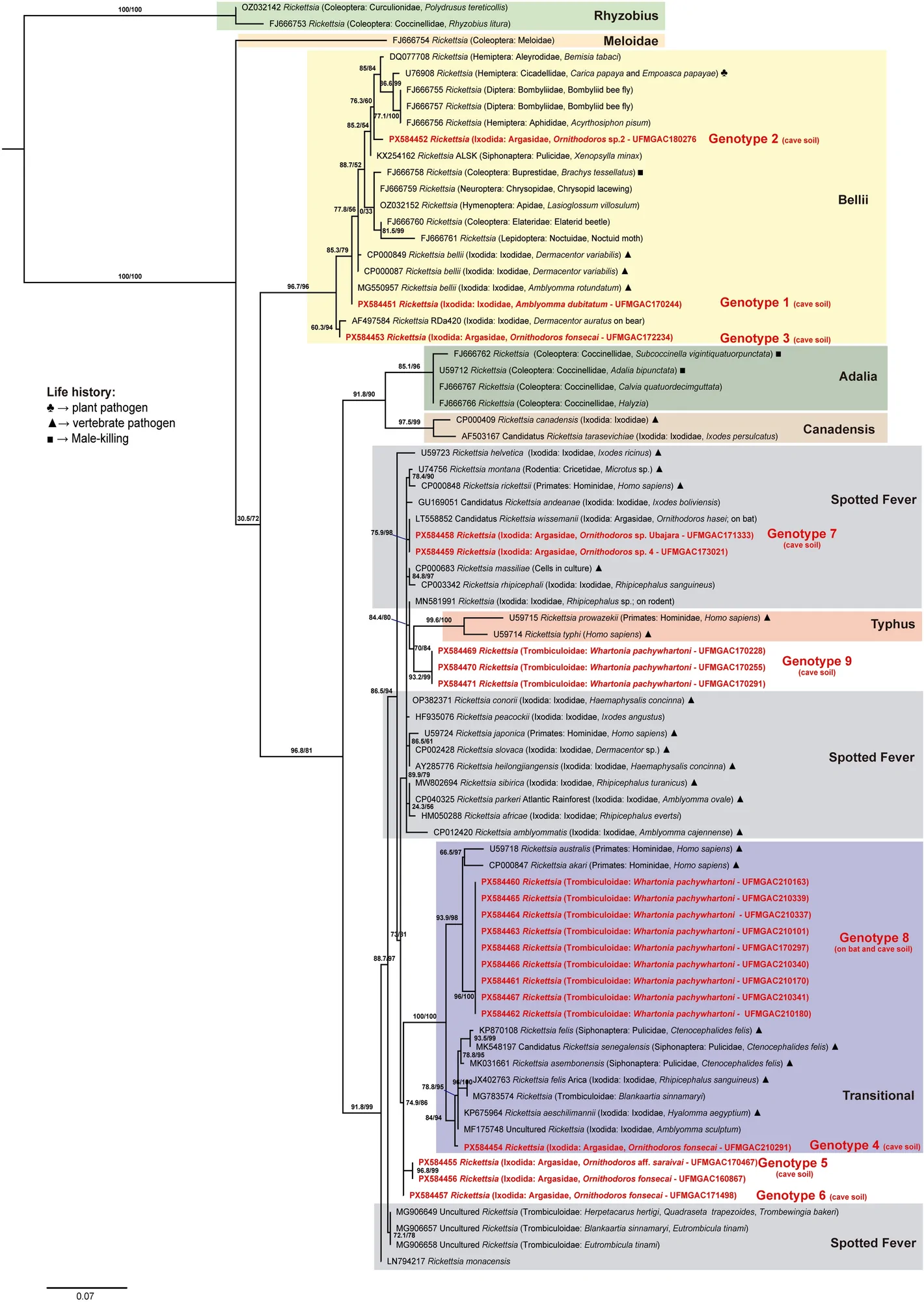
Maximum likelihood phylogenetic tree based on nucleotide sequences of citrate synthase (*gltA*) of *Rickettsia* spp. Main phylogenetic and serological groups are shaded. Values associated with branches are SHlike approximate likelihood ratio test (SH-aLRT) and UltraFast Bootstrap (UFBoot), respectively, both calculated with 1000 replicates. Newly sequenced terminals are in red as well the gltA genotypes as referred in the main text

Despite being only 379 bp long, our more comprehensive dataset, the *gltA* gene tree, led to a topology that roughly corresponds to the trees obtained by whole genome or multi loci analyses, as follows: (Rhyzobius, (Meloidae, (Bellii, ((Adalia, Canadensis), (Typhus, Transitional, Spotted Fever)))). The new sequences were recovered in the gene tree in three phylogroups, the Transitional (TRG), Spotted Fever (SFG) and Bellii groups (Fig. 3). Based on their DNA sequences, we group them in nine gltA genotypes, as described below and shown in Fig. 3.

**GltA genotype 1**: The single *Rickettsia* detected from a hard tick, a female identified as *Amblyomma dubitatum* (UFMG-AC 170244, GenBank PX584451), collected dwelling on a cave floor, belongs to the Bellii group. The Blastn search querying *gltA* sequences revealed 70 hits with 100% identity with *R. bellii*, detected from Ixodidae (genera *Amblyomma*, *Dermacentor*, *Ixodes*, and *Haemaphysalis*) from USA, El Salvador, Costa Rica, Colombia, Argentina and Brazil, including localities scattered across the country.

The family Argasidae harbored nine *Rickettsia* occurrences, including sequences recovered in three phylogroups (TRG, SFG, and Bellii groups).

**GltA genotype 2**: In the Bellii Group, the sequence obtained from the *Ornithodoros* sp. 2 (*O. yumatensis* + *O. brodyi* clade) female (UFMG AC 180276), collected near to the entrance of an iron ore cave in Minas Gerais state. BLASTn search recovered a 98.94% identity with the sequence identified as *Rickettsia* sp. ALSK (GenBank KX254162), obtained from the flea *Xenopsylla minax* (Siphonaptera, Pulicidae) from the Alataw Pass, Xinjiang, China.

**GltA genotype 3**: The sequence obtained from a female of *O. fonsecai* (UFMG AC 172234), from the twilight zone floor of a limestone cave in the southern portion of São Paulo state, was recovered in the Bellii Group too. It has an identity of 99.47% with *Rickettsia* sp. RDa420 (GenBank AF497584) detected in *Dermacentor auratus* removed from a bear in Thailand (Parola et al. 2003).

**GltA genotype 4**: A sequence from one female (UFMG-AC 210291, aphotic zone of a limestone cave) appear nested in the TRG (Fig. 3), with an identity of 99.47% with the sequence GenBank MF175748, from a *Amblyomma sculptum* individual collected in the southern Brazilian state of Paraná, and 98.94% identity with 13 sequences identified as *Rickettsia asembonensis*, eleven obtained from *Ctenocephalides felis* associated to dogs, nine in the Brazilian amazonian state of Rondônia and two from Costa Rica, a sequence obtained from *C. canis* in Kenya, and a sequence from a human patient from Malaysia.

**GltA genotype 5**: The *Rickettsia* sequences from two individuals (one *O. fonsecai* and one *O.* aff. *saraivai*) grouped in a basal clade among TRG. Obtained in Caves from Minas Gerais as follows: one female of the *O. saraivai* Clade (UFMG-AC 170467, cave soil) and a female identified as *O. fonsecai* (UFMG-AC 160867), both from twilight zone of a limestone cave. These sequences are identical and had 60 Blastn hits with sequences identified as *R. parkeri*, *R. sibirica*, *R. africae* and other unidentified bacteria, with 98.67% identity, all *Rickettsia* belonging to the SFG.

**GltA genotype 6**: The sequence from a *O. fonsecai* female (UFMG-AC 171498), from twilight zone of a limestone cave, had 52 hits with 99.20% identity with *Rickettsia* identified as *R. raoultii*, *R. conori*i, and unidentified bacteria.

**GltA genotype 7**: Other two sequences were obtained from an *Ornithodoros fonsecai* (UFMG-AC 171333) and a nymph of *O.* aff *saravai* (UFMG-AC 173021) both collected on the cave floor in the twilight zone. They were nested in the SFG and are identical to a sequence identified as ‘Candidatus *Rickettsia wissemanii*’ (GenBank LT558852) obtained from *O. hasei* ticks found on the bat *Noctilio albiventris* from French Guiana (Tahir et al 2016).

All rickettsial sequences obtained from Trombiculoidae were obtained from *Whartonia pachywhartoni* individuals.

**GltA genotype 8**: The gltA genotype with more sequences (nine terminals) comprise larvae associated to the bats *Carollia perspicillata* (UFMG AC 210163, 210,170), *Diphylla ecaudata* (UFMG AC 210180), *Mimon bennetti* (UFMG AC 210101), larvae found dwelling free on a cave floor (UFMG AC 210337, 210,339–210341) and a male (UFMG AC 170297). The Blastn hit with maximum similarity was with *Rickettsia australis* str. Cutlack (CP003338), which belongs to TRG, with 97.61% identity.

**GltA genotype 9**: The other clade included three sequences, all obtained from *W. pachywhartoni* males (UFMG AC 170228, 170,255, 170,291) dwelling on the floor of iron ore caves in the twilight and aphotic zones. Blastn searches recovered a hit with 98.14% identity with *Rickettsia* associated with *Rhipicephalus* sp. tick nymph from *Nesokia indica* (Rodentia, Muridae) in Pakistan. The following most similar hits (97.87%) are SFG members (*R. raoultii*, *R. conorii*, *R. montanensis*). However, in the phylogenetic tree, gltA genotype 9 sequences were sister to the TG clade composed by *R. prowazekii* and *R. typhi* (Fig. 3).

### Multiloci analyses

It was possible to obtain sequences from additional markers for bacteria belonging to all above delimited genotypes except for the gltA genotype 9. The concatenated alignment, after adding GenBank terminals (Supplementary Table S4) comprised 57 sequences, 2966 positions, 892 variable, and 604 parsimony-informative sites. The best-fit partitioned model, chosen according to BIC, divided the sequences into two partitions, the first with ribosomal 16S sequences and the second merging all protein coding genes. The model TPM3u+R2 (AC=CG, AG=CT, AT=GT and unequal base frequencies) was assigned to the first partition, while the model GTR+F+I+G4 (General time reversible model with unequal rates, unequal base frequencies, a proportion of invariant sites, and a gamma distribution) for the second. In MrBayes, the data was divided in the same way as in ML analyses, employing the HKY instead of TPM3u+R2 for the ribosomal 16S.

**Fig. 4:**
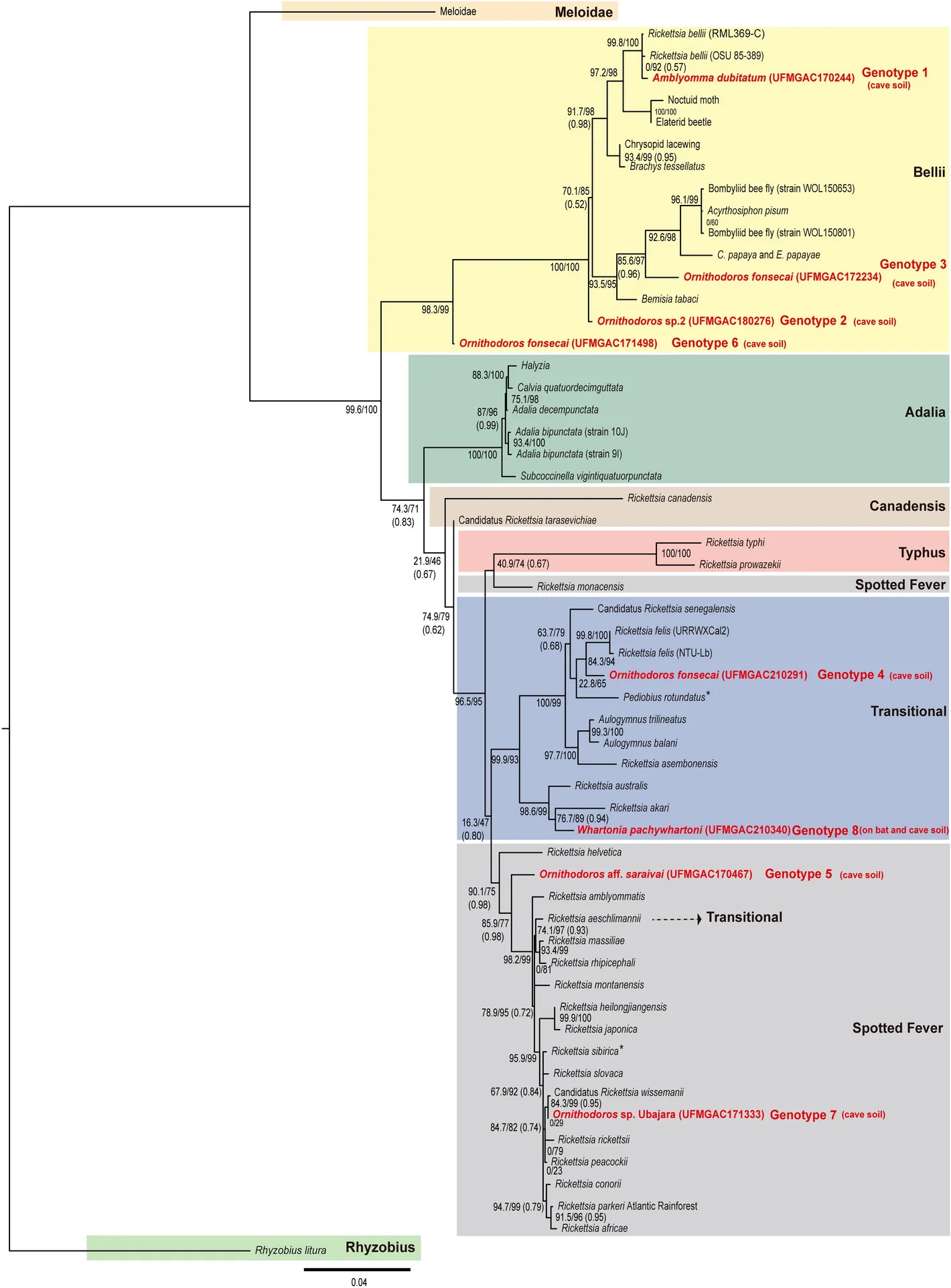
Maximum likelihood tree based on concatenated alignment of Rickettsial genes 16S, ATPase, Cytochrome c Oxidase, and Citrate Synthase. Main phylogenetic and serological groups are shaded. Values associated with branches are SHlike approximate likelihood ratio test (SH-aLRT) and UltraFast Bootstrap (UFBoot), respectively, both calculated with 1000 replicates, and Posterior Probabilities calculated in the Bayesian analysis. Posterior probabilities of 100% are not indicated. Newly sequenced terminals in red, gltA genotypes referred as in the main text

The topology of rickettsial groups recovered by the analyses was as follows: (Rhyzobius, (Meloidae, (Bellii, (Adalia, (Canadensis, (*R. monasensis* (Typhus, (Transitional, Spotted Fever)))))))). Note, however, that the Canadensis group was not supported, appearing as a paraphyletic group in the ML tree (Fig. 4).

*Rickettsia bellii* (gltA genotype 1), the only occurring in an *Amblyomma* species, could be sequenced for the four markers and grouped with the other two terminals belonging to this species. The gltA genotypes 2 and 3, as in the gltA analyses, were recovered in the Bellii Group. Whereas the support to the group is absolute, however, their internal relationship received a lower support. The endosymbiont of the *Ornithodoros* sp. 2 (UFMG AC 180276) *Rickettsia*, the gltA genotype 2, was recovered as a basal branch in the Bellii Group, and the placement of *O. fonsecai* (UFMG AC 172234) in a clade with Bombyiid bee fly, *Acyrthosiphon* and *Rickettisia* associated with *Carica papaya* and *Empoasca papayae* received a support of UFBoot = 97, SH-aLRT = 85.6, and PP = 0.96. In addition to gltA genotypes 1–3, already recovered in Belli group in single marker analyses, the genotype 6 moved in multiloci analyses, to a sister group position to this group.

The gltA genotype 4, from another *O. fonsecai* specimen (UFMG AC 210291), was recovered in a well-supported clade along *R. felis* (UFBoot = 94, SH-aLRT =84.3, and PP = 1.00). The gltA genotype 8, associated with nine specimens of *W. pachywhartoni*, was recovered in a clade with *R. akari* with considerable support (UFBoot = 89, SH-aLRT =76.7, and PP = 0.94).

The remaining genotypes were associated with the SFG, which recovered a support of UFBoot = 75, SH-aLRT = 90.1, and PP = 0.98. The gltA genotype 5 is the second branch in the SFG tree, whereas gltA genotype 7 grouped in a clade that received a good support (UFBoot = 99, SH-aLRT =84.3, and PP = 0.95), with ‘Candidatus *R. wissemanii’*.

## Discussion

### Retrieving molecular data from natural history collections

Despite growing concerns about making genetic information available for future research, curators still have to deal with specimens collected and processed solely for morphological identification. For example, Brazilian environmental laws, like those of other countries, require Environmental Impact Assessment (EIA) for economic activities such as mining, urban development, and agriculture (Morgan 2012), which includes conducting biodiversity surveys to estimate environmental impacts and indicate mitigation measures. Surveys associated with EIA result in a large number of specimens to be kept in natural history collections, often originating from natural communities destroyed by licensed economic activities. Those EIA preserve exemplars in alcohol 70% at room temperature during sorting and identification, being handled to their definitive archival condition after some months to years, hence being kept meanwhile in conditions suboptimal for DNA preservation.

Despite that many samples had their DNA degraded due the storage conditions prior its inclusion in the CCT-UFMG Acarological collection, hence precluding amplification and sequencing, this study shows that a great deal of bacterial diversity still may be retrieved from taxonomical collections, integrating geographical information, ectoparasites taxonomy and, in the case of ticks and chiggers, vertebrate sources. The microbiome of insects include not just potential pathogens, but also can have profound effects on their hosts, from negative interactions in the form of pathogenesis and parasitism to highly beneficial associations in the form of nutrient provisioning and immune defense (Short et al. 2018). It is reasonable suppose that the same is true for mites and similar to entomological collections the effort in sequencing even historical samples may lead to relevant contributions to the understanding of their microbiomes, since stable associations were already found among *Flavobacterium* and spider mites (Zhu et al. 2020), and *Clostridia* with Phytoseiidae (Yan et al. 2024).

### *Integrative taxonom*y* of ticks*

Even though not being its main focus, this study could bring some new occurrences of *Ornithodoros* species and possible new species.

In the case of *O. fonsecai,* it is remarkable that both haplogroups detected in Oliveira et al (2024) could be found in this study, up to now congruent to one haplogroup associated to localities more arid than to the other. A possible direction for future investigation concerning this species is testing if the distribution of those haplogroups corresponds to ongoing local adaptation.

The still unnamed species “*Ornithodoros* sp Ubajara” recorded by Jorge et al (2022), proved to be abundant in caves around southern portion of Espinhaço Range (Lagoa Santa and Pedro Leopoldo), including larvae parasitizing the nectivorous bat *Glossophaga soricina* (Phyllostomidae). Despite the specimens reported herewith came from caves in transitional areas between the Atlantic Forest and Cerrado, apart almost 1800 km from the original records, the p-distances and PTP analyses left no doubts about its identity.

The sequences grouped with *O. saraivai* warrants further investigation, since they exhibit considerable divergence, and the ticks reported here may constitute a species complex. Finally, some specimens may belong to undescribed species, such as the nymph UFMG AC 180276, collected near the entrance of a cave in the Santa Maria de Itabira municipality, Minas Gerais state. All these novelties related to Argasidae are not unexpected, since many new species were recently described (Dantas-Torres et al. 2019).

### Rickettsia *diversity*

The phylogenetic diversity found in the *Rickettsia* sequenced herewith likely imply a similar ecological diversity. We suggest that a possible explanation may be its association with bats and caves used as roosts by bats. This hypothesis must be further investigated by systematic sampling of fresh material.

Among detected *Rickettsia* spp., only *Rickettsia bellii* was found associated with an Ixodid, *Amblyomma dubitatum*, primarily a capybara (*Hydrochoerus hydrochaeris*) ectoparasite, but already found parasitizing the bat *Glossophaga soricina* (Phyllostomidae) (Nava et al. 2010). *Rickettsia bellii* presents distinct lineages for North and South America (Krawczak et al 2018), and the *gltA* sequence obtained here groups with other South-American bacteria. It is regarded as nonpathogenic for vertebrates, despite experimental studies showing that inoculation of this *Rickettsia* produces eschars in rabbits and guinea pigs (Ogata et al 2006).

The other arthropod hosts for *Rickettisia* spp in this study, the genera *Ornithodoros* and *Whartonia*, are primarily or at least putative bat ectoparasites that spend some time dwelling on the cave floor. Hence, most of rickettsial diversity found in this study came from arthropods that have stages on and off bat hosts, being integrated during part of their lives in the ecology of the cave soil, including examples of hyperparasitism by other mites, such as Erythraeidae larvae (e.g. Bassini-Silva et al 2019).

For instance, *Rickettsia* is recorded from blood and tissue samples of bats, including disparate world regions as in South Africa and Swaziland, where a lineage related *R. conorii* was detected (Dietrich et al. 2016), Romania, where a high prevalence of *R. monacensis* was detected in insectivorous bats (Matei et al. 2021), China, where it was detected the presence of several SFG agents (Zhao et al 2020). It is also often detected in bats ectoparasites such as ticks (especially Argasidae), mites, and flies (reviewed in Matei et al. 2021), where the SFG and TRG were detected, the later with sequences related to *R. felis*.

In our study, along occurrences related to bacteria already found in bat ectoparasites, as ‘Candidatus *Rickettsia wissemanii*’ found in *Ornithodoros hasei* from French Guiana, Brazil and Argentina (Tahir et al. 2016; Colombo et al. 2020; Luz et al. 2019), above referred as gltA genotype 7, to *R. felis* (gltA genotype 4) and included in the SFG (gltA genotype 5 and likely gltA genotype 9), we recorded some lineages that seems to be found for the first time harbored by bat ectoparasites. It is the case for gltA genotypes 2 and 3, both nested in the Bellii group, and in the multiloci analyses with terminals with phenotypes so diverse as male-killing and plant pathogens (Hurst and Jiggins 2000; Davis et al. 1998). It is also the case for gltA genotype 8, found in chiggers. It nests in the TRG group along *R. australis* and *R. akari,* both of medical relevance, being the causative agents of the Queensland Tick Typhus and rickettsialpox (Stewart et al 2017; Denison et al 2014).

### Ornithodoros *in caves as a pathogen vector for humans*

Soft ticks are mostly reported as human parasites by benefiting from the conditions provided by the human houses and synanthropic fauna, including bats (Bermúdez et al. 2021). However, the increasing use of caves as recreational and touristic spots, or its use in economical activities like mining, bring humans in contact to the subterranean fauna, and may make biting of soft ticks more common. Jorge et al (2022) reported biting by *O. cavernicolous* and *O. fonsecai*, the later already reported to occur in other studies (Labruna and Venzal 2009; Santiago et al. 2019). As in this article we found four out nine gltA genotypes of *Rickettsia* in the latter species, warranting the need of a close inspection of its potential role in the appearance of emergent rickettsiosis.

Finally, it is noteworthy that research on *Rickettsia* spp. in ticks has been strongly focused on hard ticks of the Ixodidae family. For example, until 2021, there were 599 records of 31 species of *Rickettsia* reported from 50 species of Ixodidae ticks in the neotropical region (Estrada-Peña et al. 2021). On the other hand, until the present study, there were only 15 records of *Rickettsia* in ticks of the Argasidae family in the neotropics, as follows: *R. lusitaniae* in *O. yumatensis* and *Ornithodoros* sp. from Mexico (Sánchez-Montes et al 2016; Hornok et al 2019), *R. bellii* in *O. talaje* from Mexico (Guzmán-Cornejo et al. 2022), *R. felis* in *O. puertoricensis* from Mexico (Ballados-González et al. 2023), ‘Candidatus *Rickettsia nicoyana*’ in *O. knoxjonesi* from Costa Rica (Moreira-Soto et al 2017), *Rickettsia* sp. in *O. rietcorreai* from Brazil (Muñoz-Leal et al. 2019a), *Rickettsia* sp. in *O.* cf. *mimon* from Brazil (Dantas-Torres et al. 2022), *Rickettsia* sp. strain Itinguçú in *O. faccinii* from Brazil (Peixoto et al. 2021), ‘Candidatus *Rickettsia wissemanii*’ in *O. hasei* from Brazil, French Guiana and Argentina (Tahir et al. 2016; Colombo et al. 2020; Luz et al. 2019; Dornelas Júnior et al. 2025), *Rickettsia* sp. in *O. amblus* from Peru and *O. peruvianus* from Chile (Duron et al. 2017), and *Rickettsia* sp. in *Ornithodoros* sp. from Chile (Muñoz-Leal et al. 2019b). Undoubtedly, the present study shows a diversity of *Rickettsia* spp. in Argasidae never before reported in a single study, contributing significantly to a better understanding of this important group of tick-borne bacteria, which has been neglected in argasids.

## Supplementary Information

Below is the link to the electronic supplementary material.

Supplementary file1 (DOCX 5550 KB)

Supplementary file2 (TIF 37762 KB) Fig. S1. Screen shots from the Tracer 1.7 program, showing values of ESS. A – gltA only analyses. B – Multiloci analyses

Supplementary file3 (TIF 10181 KB) Fig. S2. Bayesian majority rule consensus tree from trees sampled during the stationary Markov Chain, based on nucleotide sequences of citrate synthase (gltA) of *Rickettsia* spp. Values associated with branches are Posterior Probabilities

Supplementary file4 (TIF 5683 KB) Fig. S3. Bayesian majority rule consensus tree from trees sampled during the stationary Markov Chain, based on nucleotide sequences of citrate synthase (gltA), 16SrDNA (Small Subunit Ribosomal DNA), atpA (ATP synthase subunit alpha), and coxA (cytochrome C oxidase subunit I) of *Rickettsia* spp. Values associated with branches are Posterior Probabilities.

## Data Availability

Sequences were deposited in GenBank. Accession numbers are refereed in the supplementary tables. Voucher material is deposited in the Acarological Collection at Centro de Coleções Taxonômicas da UFMG (UFMG AC).
