# Supplementary material for: Molecular detection of Rickettsia spp. in ticks (Ixodida: Argasidae and Ixodidae) and mites (Trombiculoidae) from a Brazilian taxonomic collection
Source: Exp Appl Acarol. 2026 Oct 28;97(4):57. doi: 10.1007/s10493-026-01200-4 (PMC13719841; doi:10.1007/s10493-026-01200-4)
Supplement: Supplementary file 1 [file 10493_2026_1200_MOESM1_ESM.docx]

**List of collection numbers of assayed individuals. Details may be found at GBIF collection database, https://www.gbif.org/dataset/2c8232a7-7886-45e5-85b6-9920fb65a2d6**

**Ixodida:**

20211 221253 221254 210050 210212 210289 210291 210391 180183 180276 170067 170068 170174 170242 170243 170467 171293 171333 171343 171470 171495 171497 171498 171631 171662 172072 172220 172233 172234 172251 172252 172886 173020 173021 173843 173848 160197 160202 160203 160213 160220 160222 160298 160303 160304 160307 160312 160313 160314 160316 160319 160320 160321 160323 160325 160328 160329 160331 160334 160335 160336 160339 160340 160341 160343 160348 160349 160350 160867 161188 161190 151019 151022 151023 151036 151041 151043 151045 151049 151051 151052 151062 151081 151083 151084 151085 151086 151158 151511 172073 220034 210052 210097 210203 210207 210285 180090 180353 170055 170057 170060 170061 170062 170064 170065 170066 170070 170231 170244 170385 170468 170483 170484 170485 170501 170507 170540 170577 170989 171024 171025 171085 171086 171197 171198 171199 171200 171496 171640 172253 172906 173792 174530 174539 174540 174541 174544 174546 174549 161071 151034 151038 151044 151056 151057 151065 173837 173845 171476 171494 171547 171548 171576 171588 173810 160198 160227 220617

**Trombiculoidae**:

1300050 1300419 1300419 1300432 1400787 1400812 1400813 1400814 1400815 1400816 151121 160062 160070 160177 160187 160193 160212 160216 160219 160301 160729 160737 160738 160747 161055 161056 170002 170017 170018 170035 170228 170229 170230 170254 170255 170258 170263 170270 170281 170288 170289 170291 170295 170297 170325 170326 170327 170329 170435 170452 170465 170927 170977 171015 171037 171039 171111 171174 171175 171471 171473 172117 172125 173926 174465 174466 174467 174508 174509 174578 180252 180266 180490 210069 210070 210071 210080 210081 210082 210084 210085 210086 210098 210101 210136 210137 210138 210139 210150 210156 210157 210158 210161 210162 210163 210170 210172 210173 210174 210180 210182 210190 210191 210193 210194 210195 210232 210238 210239 210240 210241 210243 210244 210246 210248 210249 210250 210251 210306 210307 210327 210331 210332 210333 210337 210339 210340 210341 210346 210347 210348 210349 210351 210352 210362 220001 220005 220518 221258 221264 210303 180382 180396 180420 1300180 1300576 1300577 1300578 1300617 160114 160211 160218 160297 160305 160306 160309 160324 160731 160732 160734 160735 160736 160864 161085 161094 161125 161126 161127 161128 161171 161173 161174 170021 170215 170259 170280 170294 170445 170602 170603 170604 170605 170608 170609 170864 170865 170867 170868 170869 171051 171081 171082 171108 171472 171572 172130 174133 174475 174476 174478 210018 210032 210068 210393 220010

**Table S1** Sample data of Ixodida (Argasidae and Ixodidae) tested for the *gltA* gene, including taxonomic identification, collection locality and host, and voucher numbers of the voucher material housed at UFMG Acarological Collection (UFMG AC). Stage/sex: L, larva; N, Nymph. Host or substrate: CS, Cave soil; A, aphotic; E, entrance; T, twilight; PESNT, Parque Estadual Serra Nova e Talhado; PES, Parque Estadual do Sumidouro. 16S rDNA, Large Subunit Mitochondrial Ribosomal DNA; COI, Mitochondrial Cytochrome C Oxidase subunit I; *gltA*, Citrate Synthase.

| Sampling data | Voucher | Stage/sex | Host or substrate | Coordinates | | GenBank Acession # | | |
| --- | --- | --- | --- | --- | --- | --- | --- | --- |
|  |  |  |  | Lat. | Log | 16S | COI | gltA |
| Family Argasidae Koch, 1844 | | | | | | | | |
| *Ornithodoros cavernicolous* Dantas-Torres, Venzal & Labruna, 2012 | | | | | | | | |
| Cave Gruta da Chácara, PESNT, Rio Pardo de Minas, MG, 20.Dec.2021 | 220211 | L | *G. soricina* | -15.6758 | -42.7295 | PX625609 | PZ374644 | – |
| Cave Lapa Grande, PELG, Montes Claros, MG,14 Dec. 2021 | 221254 | L | *M. bennetti* | -16.7067 | -43.9549 | NA | PZ374645 | – |
| Cave HCM 41, Pedro Leopoldo, MG, 09 – 08 Nov. 2016 | 172073 | ♀ | *CS,T* | -19.5750 | -44.0198 | NA | PZ374646 | – |
| Cave HCM-0041, Pedro Leopoldo, MG, 08 Jul. 2017 | 172886 | ♂ | *CS,E* | -19.5750 | -44.0199 | NA | PZ374647 | – |
| *Ornithodoros fonsecai* (Labruna & Venzal, 2009) | | | | | | | | |
| Cave Lapa das Pacas, PES, Lagoa santa, MG, 12 Aug. 2021 | 210291 | ♀ | CS,A | -19.5607 | -43.9605 | PX625610 | NA | PX584454 |
| Cave HOLC-0102, Pedro Leopoldo, MG, 21 Nov. – 02 Dec. 2016 | 171497 | N | CS,E | -19.5978 | -44.0190 | PX625611 | PZ374648 | – |
| Cave HOLC-0102, Pedro Leopoldo, MG, 02–14.Jun.2016 | 171498 | ♀ | CS,T | -19.5978 | -44.0191 | PX625612 | NA | PX584457 |
| Cave HOLC-097/90, Pedro Leopoldo, MG, 27 Jun. – 01 Jul. 2016 | 171470 | ♂ | CS | -19.6029 | -44.0120 | PX625613 | NA | – |
| Cave Baixada das Crioulas, Itambé do Mato Dentro, MG, 23 – 24 Sep. 2015 | 160867 | ♀ | CS,T | -19.4075 | -43.3575 | PX625614 | NA | PX584456 |
| Cave Lapa Sem Fim, Luislândia, MG, 10 – 27 Apr. 2015 | 160197 | ♀ | CS | -16.1489 | -44.6275 | PX625615 | NA | – |
| Cave Lapa Sem Fim, Luislândia, MG, 10 – 27 Apr. 2015 | 160298 | ♀ | CS | -16.1489 | -44.6275 | PX625616 | NA | – |
| Cave Lapa Sem Fim, Luislândia, MG, 10 – 27 Apr. 2015 | 160303 | N | CS | -16.1489 | -44.6275 | PX625617 | NA | – |
| Cave Lapa Sem Fim, Luislândia, MG, 10 – 27 Apr. 2015 | 160307 | N | CS | -16.1489 | -44.6275 | PX625618 | NA | – |
| Cave Lapa Sem Fim, Luislândia, MG, 10 – 27 Apr. 2015 | 160312 | ♀ | CS | -16.1489 | -44.6275 | PX625619 | NA | – |
| Cave Lapa Sem Fim, Luislândia, MG, 10 – 27 Apr. 2015 | 160313 | N | CS | -16.1489 | -44.6275 | PX625620 | NA | – |
| Cave MTD-09, Ribeira, SP, 08-20 Mar. 2016 | 172233 | ♂ | CS,T | -24.6388 | -48.9621 | NA | PZ374649 | – |
| Cave MTD-09, Ribeira, SP, 08-20 Mar. 2016 | 172234 | ♀ | CS,T | -24.6388 | -48.9621 | NA | PZ374650 | PX584453 |
| Clade *Ornithodoros saraivai* Muñoz-Leal & Labruna, 2017 | | | | | | | | |
| Cave PDR-0073, Congonhas, MG, 18 – 20 Sep. 2018 | 180183 | N | CS, E | -20.4692 | -43.9032 | PX625621 | NA | – |
| Cave VL 14, Itabirito, MG, 09 – 15 Mar. 2017 | 171662 | ♀ | CS | -20.2946 | -43.9467 | PX625622 | NA | + |
| Cave CSS-0098, Conceição do Mato Dentro, MG, 22 Nov. – 15 Dec. 2016 | 170467 | ♀ | CS | -18.9419 | -43.4100 | PX625623 | NA | PX584455 |
| Cave SPT-0051, Conceição do Mato Dentro, MG, 26 Jun. – 13.Oct. 2016 | 170067 | N | CS,T | -19.1675 | -43.2739 | PX625624 | PZ374651 | – |
| Cave SPT-0051, Conceição do Mato Dentro, MG, 16 – 26 Jan. 2017 | 170174 | ♀ | CS, E | -19.1675 | -43.2739 | PX625625 | PZ374652 | – |
| Cave RM-33, Nova Lima, MG, 12 Jan. 2021 | 210050 | ? | CS | -20.0349 | -43.9946 | PX625626 | NA | + |
| Cave SPT-0444, Conceição do Mato Dentro, MG, 16 – 26 Jan.2017 | 170243 | ♂ | CS, E | -19.2150 | -43.3825 | NA | PZ374661 | – |
| *Ornithodoros* sp. 2 (*O. yumatensis* + O. brodyi clade) | | | | | | | | |
| Cave CP-0004, Santa Maria de Itabira, MG, 17 Aug. 2018 | 180276 | N | CS, E | -19.3264 | -43.3123 | PX625627 | NA | PX584452 |
| *Ornithodoros* sp. 3 (*Ornithodoros* sp. Ubajara) | | | | | | | | |
| Cave HOLC-0063, Pedro Leopoldo, MG, 22 Jul. – 18 Aug. 2015 | 151049 | N | CS | -19.5711 | -44.0139 | PX625628 | PZ374653 | – |
| Cave Lapa do sumidouro, PES, Lagoa santa, MG, 10 Aug. 2021 | 220034 | N | *D. rotundus* | -19.5418 | -43.9411 | PX625629 | PZ374654 | – |
| Cave Lapa do sumidouro, PES, Lagoa santa, MG, 16 Dec. 2020 | 210097 | N | *G. soricina* | -19.5418 | -43.9411 | PX625630 | PZ374655 | – |
| Cave HOLC-0021, Pedro Leopoldo, MG, 22 Jul. – 18 Aug. 2015 | 151043 | N | CS | -19.5708 | -44.0094 | PX625631 | NA | – |
| Cave HOLC-0021, Pedro Leopoldo, MG, 04 – 20 Mar. 2015 | 151045 | ♀ | CS | -19.5708 | -44.0094 | PX625632 | NA | – |
| Cave HOLC-157, Pedro Leopoldo, MG, 14 Jun. 2016 | 171343 | N | CS, E | -19.5715 | -44.0116 | NA | PZ374656 | – |
| Cave HOLC-0139, Pedro Leopoldo, MG, 21 Nov. – 02 Dec. 2016 | 171333 | ♀ | CS,T | -19.5816 | -44.0198 | NA | PZ374657 | PX584458 |
| Cave HOLC-0138, Pedro Leopoldo, MG, 02 – 14 Jun. 2016 | 171293 | N | CS,T | -19.5816 | -44.0198 | NA | PZ374658 | – |
| Cave HOLC-0046, Pedro Leopoldo, MG, 04 – 20 Mar. 2015 | 151051 | N | CS | -19.5803 | -44.0183 | NA | PZ374659 | – |
| Cave HOLC-0021, Pedro Leopoldo, MG, 22 Jul. – 18 Aug. 2015 | 151052 | N | CS | -19.5708 | -44.0094 | NA | PZ374660 | – |
| Cave HOLC-0046, Pedro Leopoldo, MG, 04 – 20 Mar. 2015 | 151511 | N | CS | -19.5803 | -44.0183 | PX625633 | NA | – |
| *Ornithodoros* sp. 4 | | | | | | | | |
| Cave PA-08, Janaúba, MG, Brazil, 08 Fev. 2017 | 173020 | ♂ | CS,T | -15.7244 | -43.4410 | NA | PZ374662 | – |
| Cave PA-08, Janaúba, MG, Brazil, 08 Fev. 2017 | 173021 | N | CS,T | -15.7244 | -43.4410 | NA | PZ374663 | PX584459 |
| Family Ixodidae Koch, 1844 | | | | | | | | |
| *Amblyomma nodosum* Neumann, 1899 | | | | | | | | |
| Cave CSS-0099, Conceição do Mato Dentro, MG, 31 May – 12 Jun. 2016 | 170385 | ♀ | CS, E | -18.9450 | -43.4056 | PX529393 | PZ374664 | – |
| *Amblyomma dubitatum* Neumann 1899 | | | | | | | | |
| Cave SPT-0583, Conceição do Mato Dentro, MG, 16 – 26 Jan. 2017 | 170244 | ♀ | CS | -19.1731 | -43.2644 | NA | PZ374665 | PX584451 |
| *Amblyomma brasiliense* Aragão, 1908 | | | | | | | | |
| Cave GAND-127, Rio Acima, MG, 14 Jul. – 18 Sep. 2016 | 170577 | N | CS, T | -20.0530 | -43.6614 | PX529394 | PZ374666 | – |
| Cave GAND-110, Rio Acima, MG, 14 Jul. – 18 Sep. 2016 | 171024 | N | CS | -20.0670 | -43.6694 | PX529395 | NA | – |
| Cave SPT-0154, Conceição do Mato Dentro, MG, 26 Jun. – 13 Oct. 2016 | 170057 | N | CS | -19.1644 | -43.2781 | NA | PZ374667 | – |
| *Amblyomma pacae* Aragão, 1911 | | | | | | | | |
| Cave SPT-0585, Conceição do Mato Dentro, MG, 16 – 26 Jan. 2017 | 170231 | ♀ | CS | -19.1744 | -43.2647 | PX529396 | NA | – |
| *Amblyomma ovale* Koch, 1844 | | | | | | | | |
| Cave HOLC-00103, Pedro Leopoldo, MG, 21 Nov. – 02 Dec. 2016 | 171496 | ♀ | CS, E | -19.6800 | -44.0109 | PX529397 | PZ374668 | – |
| Cave HOLC-0040, Pedro Leopoldo, MG, 22 Jul. – 18 Aug. 2015 | 151044 | ♀ | CS | -19.5714 | -44.0142 | PX529398 | NA | – |
| Cave MTD-12, Ribeira, SP, 08 – 20 Mar. 2016 | 172253 | ♀ | CS, T | -24.6433 | -48.9589 | NA | PZ374669 | – |
| *Amblyomma sculptum* Berlese, 1888 | | | | | | | | |
| Cave SPT-0161, Conceição do Mato Dentro, MG, 26 Jul. – 13 Oct. 2016 | 170064 | N | CS, E | -19.1608 | -43.2775 | PX529399 | PZ374670 | – |
| Cave MTD-0008, Mariana, MG, Brazil, 11-13.IX.2018 | 174546 | N | CS, E | -20,1668 | -43,3856 | PX529400 | NA | – |
| Cave HOLC-0118, Pedro Leopoldo, MG, 04 – 20 Mar. 2015 | 151038 | ♀ | CS | -19.5769 | -44.0206 | PX529401 | NA | – |
| Cave SPT-0245, Conceição do Mato Dentro, MG, 26 Jul. – 05 Aug. 2016 | 170483 | N | CS | -19.0914 | -43.3625 | PX529402 | NA | – |
| Cave CCB-0052, Mariana, MG, 11-13 Sep. 2018 | 174549 | ♀ | CS, T | -20.1664 | -43.3833 | PX529403 | PZ374671 | – |
| Cave SPT-0270, Conceição do Mato Dentro, MG, 29 Nov. – 16 Dec. 2016 | 170055 | ♀ | CS, E | -19.0867 | -47.1644 | PX529404 | NA | – |
| Cave SPT-0503, Conceição do Mato Dentro, MG, 10 – 15 Jan. 2017 | 171576 | ? | CS | -19.2185 | -43.3862 | NA | PZ374672 | – |
| Cave SPT-0233, Conceição do Mato Dentro, MG, 26 Jul. – 13 Oct. 2016 | 170507 | N | CS, E | -19.0919 | -43.3689 | PX529405 | NA | – |
| *Amblyomma cajennense* Fabricius, 1787 | | | | | | | | |
| Cave PS-00015, Marabá, PA, 01 Aug. 2018 | 180090 | ♀ | CS, E | -5.7884 | -50.4988 | PX529406 | NA | – |

**Table S2** Sample data of chiggers belonging to the genus *Whartonia* Ewing, 1944 including taxonomic identification, collection locality and host, and voucher numbers of the voucher material housed at UFMG Acarological Collection (UFMG AC). Stage/sex: L, larva; DN, Deutonymph. Host or substrate: CS, Cave soil; A, aphotic; E, entrance; T, twilight. 28S, Large Subunit Nuclear Ribosomal DNA; COI, Mitochondrial Cytochrome C Oxidase subunit I; *gltA*, Citrate Synthase.

| Sampling data | Voucher | Stage/sex | Host or substrate | Coordinates | | GenBank Acession # | | |
| --- | --- | --- | --- | --- | --- | --- | --- | --- |
|  |  |  |  | Lat. | Log | 28S rDNA | COI | gltA |
| *Whartonia pachywhartoni* Vercammen-Grandjean, 1966 | | | | | | | | |
| PESRM, Belo Horizonte, MG, 02 Jun. 2013 | 1300432 | L | *C. perspicillata* | -20.0347 | -43.9945 | NA | ON145636 | + |
| Cave CSS 15, Conceição do Mato Dentro, MG, 26 – 29 Jun. 2016 | 160177 | ♀ | CS, T. | -18.9106 | -43.4286 | NA | ON145625 | – |
| Cave Lapa Sem Fim, Luislândia, MG, 10 – 27 Apr. 2015 | 160212 | ♀ | CS | -16.1489 | -44.6275 | ON146225 | ON145627 | – |
| Cave SPT-0490, Conceição do Mato Dentro, MG, 26 Jul. – 13 Oct. 2016 | 161056 | ♀ | CS | -19.2183 | -43.3864 | NA | ON145648 |  |
| Cave SPT-0081, Serpentina Ridge, Conceição do Mato Dentro, MG, 26 Jun. – 13 Oct. 2016 | 170002 | DN | CS, A. | -19.1664 | -43.2711 | NA | ON145637 | + |
| Cave SPT-0081, Serpentina Ridge, Conceição do Mato Dentro, MG, 26 Jun. – 13 Oct. 2016 | 170018 | ♀ | CS | -19.2214 | -43.3903 | NA | ON145654 | – |
| Cave SPT-0585, Serpentina Ridge, Conceição do Mato Dentro, MG 16 – 26 Jan. 2017 | 170228 | ♂ | CS | -19.1744 | -43.2647 | NA | ON145642 | PX584469 |
| Cave SPT-0585, Serpentina Ridge, Conceição do Mato Dentro, MG 16 – 26 Jan. 2017 | 170230 | ♀ | CS | -19.1744 | -43.2647 | NA | ON145670 | – |
| Cave SPT-0316, Serpentina Ridge, Conceição do Mato Dentro, MG 16 – 26 Jan. 2017 | 170255 | ♂ | CS, A | -19.2214 | -43.3903 | NA | ON145653 | PX584470 |
| Cave SPT-0160, Serpentina Ridge, Conceição do Mato Dentro, MG 16 – 26 Jan. 2017 | 170258 | ♀ | CS, A | -19.1647 | -43.2789 | NA | ON145652 | – |
| Cave SPT-0151, Serpentina Ridge, Conceição do Mato Dentro, MG, 29 Nov. – 16 Dec. 2016 | 170270 | ♀ | CS | -19.0925 | -43.3644 | NA | ON145641 | – |
| Cave SPT-0131, Serpentina Ridge, Conceição do Mato Dentro, MG, 16 – 26 Jan. 2017 | 170281 | ♀ | CS, A | -19.0850 | -47.1669 | NA | ON145645 | – |
| Cave SPT-0316, S. Serpentina, Conceição do Mato Dentro, MG, 16 – 26 Jan. 2017 | 170288 | ♀ | CS, A | -19.2214 | -43.3903 | NA | ON145628 | – |
| Cave SPT-0316, S. Serpentina, Conceição do Mato Dentro, MG, 16 – 26 Jan. 2017 | 170289 | ♂ | CS, A | -19.2214 | -43.3903 | NA | ON145651 | – |
| Cave SPT-0316, S. Serpentina, Conceição do Mato Dentro, MG, 16 – 26 Jan. 2017 | 170291 | ♂ | CS, T. | -19.2214 | -43.3903 | ON146214 | ON145650 | PX584471 |
| Cave SPT-0585, S. Serpentina, Conceição do Mato Dentro, MG, 16 – 26 Jan. 2017 | 170325 | ♂ | CS | -19.1744 | -43.2647 | NA | ON145640 | – |
| Cave SPT-0585, S. Serpentina, Conceição do Mato Dentro, MG, 16 – 26 Jan. 2017 | 170326 | DN | CS | -19.1744 | -43.2647 | NA | ON145638 | – |
| Cave MCFIN-0005, Itambé do Mato Dentro, MG, 26 Jun. – 06 Jul. 2018 | 180252 | ♂ | CS, A | -19.3318 | -43.3076 | NA | ON145644 | – |
| Cave MGB-0063, Ouro Preto, MG, 03 – 06 Nov. 2018 | 180490 | ♂ | CS, A | -20.4437 | -43.7654 | NA | ON145626 | + |
| Cave Simmons, São Gonçalo do Rio Abaixo, MG, Oct. 2016 | 210081 | ♀ | CS | -19.8986 | -43.4728 | NA | ON145647 | – |
| Cave Nadinho, Pains, MG, 11 Aug. 2020 | 210157 | L | *D. rotundus* | -20.3678 | 45.6573 | ON146215 | ON145657 | – |
| Cave Coruja, Doresópolis, MG, 05 Aug. 2020 | 210161 | L | *D. rotundus* | -20.2938 | -45.8539 | ON146220 | ON145630 | – |
| Cave Brega, Pains, MG, 03 Aug. 2020 | 210162 | L | *C. perspicillata* | -20.4174 | -45.7718 | ON146212 | ON145656 | – |
| Cave Olhos d'água, Pains, MG, 13 Mai. 2020 | 210173 | L | *S. lilium* | -20.3116 | -45.6847 | ON146226 | ON145635 | – |
| Cave Olhos d'água, Pains, MG, 13 Mai. 2020 | 210174 | L | *P. lineatus* | -20.3116 | -45.6847 | ON146213 | ON145646 | + |
| Cave Paca, Formiga, MG, 05 Sep. 2020 | 210180 | L | *D. ecaudata* | -20.4743 | -45.6595 | ON146216 | ON145629 | PX584462 |
| Cave Paca, Formiga, MG, 05 Sep. 2020 | 210190 | L | *A. caudifer* | -20.4743 | -45.6595 | ON146224 | ON145658 | + |
| Cave Paca, Formiga, MG, 04 Jan. 2020 | 210194 | L | *D. ecaudata* | -20.4743 | -45.6595 | ON146217 | ON145660 | – |
| Gruta Olhos d'água, Pains, MG, 31 Mar. 2020 | 210195 | L | *C. perspicillata* | -20.3116 | -45.6847 | ON146227 | ON145659 | + |
| Cave Paca, Formiga, MG, 05 Sep. 2020 | 210232 | L | *C. perspicillata* | -20.4743 | -45.6595 | ON146221 | ON145661 | + |
| Cave Paca, Formiga, MG, 05 Sep. 2020 | 210241 | L | *M. bennettii* | -20.4743 | -45.6595 | ON146222 | ON145662 | – |
| Cave Coruja, Doresópolis, MG, 05 Mai. 2020 | 210243 | L | *D. rotundus* | -20.2934 | -45.8535 | NA | ON145663 | – |
| Cave Coruja, Doresópolis, MG, 05 Mai. 2020 | 210244 | L | *D. rotundus* | -20.2934 | -45.8535 | NA | ON145664 | – |
| Cave Coruja, Doresópolis, MG, 05 Mai. 2020 | 210246 | L | *D. rotundus* | -20.2934 | -45.8535 | ON146223 | ON145665 | – |
| Cave Paca, Formiga, MG, 04 Jan. 2020 | 210248 | L | *D. ecaudata* | -20.4743 | -45.6595 | ON146218 | ON145666 | – |
| Cave RM-38, PESRM, Nova Lima, MG, 14 Jan. 2021 | 210251 | ♀ | CS, A | -20.0135 | -43.9787 | NA | ON145631 | – |
| Cave SPT-0131, S. Serpentina, Conceição do Mato Dentro, MG, 16–26 Jan. 2017 | 170297 | ♂ | CS, T | -19.0907 | -43.3654 | NA | ON145633 | PX584468 |
| PES, Lagoa Santa, MG, 16 Dec. 2020 | 210101 | L | *M. bennetti* | -19.5418 | -43.9411 | PX620026 | NA | PX584463 |
| Cave Gruta do Brega, Pains, MG, 08 Mar. 2020 | 210163 | L | *C. perspicillata* | -20.4174 | -45.7718 | PX620025 | NA | PX584460 |
| Cave Gruta do Brega, Pains, MG, 08 Mar. 2020 | 210170 | L | *C. perspicillata* | -20.4174 | -45.7718 | NA | PX532403 | PX584461 |
| Cave RM 33, PERM, Nova Lima, MG, 24 Aug. 2021 | 210337 | L | *C. perspicillata* | -20.0350 | -43.9947 | PX620027 | NA | PX584464 |
| Cave RM 33, PERM, Nova Lima, MG, 24 Aug. 2021 | 210339 | L | *C. perspicillata* | -20.0350 | -43.9947 | PX620028 | NA | PX584465 |
| Cave RM 33, PERM, Nova Lima, MG, 24 Aug. 2021 | 210340 | L | *C. perspicillata* | -20.0350 | -43.9947 | PX620029 | NA | PX584466 |
| Cave RM 33, PERM, Nova Lima, MG, 24 Aug. 2021 | 210341 | L | *C. perspicillata* | -20.0350 | -43.9947 | NA | NA | PX584467 |
| *Whartonia nudosetosa* (Wharton, 1938) | | | | | | | | |
| Cave GEM-1614, Canaã dos Carajás, PA 19 – 24 Nov. 2014 | 160070 | DN | CS | -6.4561 | -50.2419 | – | ON145620 | – |
| Cave GEM-1614, Canaã dos Carajás, PA, 06 – 19 Nov. 2015 | 160193 | ♀ | CS | -6.4561 | -50.2419 | ON146207 | ON145621 | – |
| Cave Lapa Sem Fim, Luislândia, MG, 10 – 27 Apr. 2015 | 160216 | ♀ | CS | -16.1489 | -44.6275 | ON146210 | ON145623 | – |
| Cave Lapa Sem Fim, Luislândia, MG, 10 – 27 Apr. 2015 | 160219 | ♀ | CS | -16.1489 | -44.6275 | ON146209 | ON145622 | – |
| Cave SPT-0585, Serpentina Ridge, Conceição do Mato Dentro, MG 16 – 26 Jan. 2017 | 170263 | ♂ | CS, T | -19.2219 | -43.3953 | NA | ON145624 | – |
| Cave GEM-1614, Canaã dos Carajás, PA, 02 – 13 Mai. 2016 | 172125 | ♀ | CS | -6.4560 | -50.2421 | NA | ON145655 | – |
| Cave Planaltina (Sitio Ecológico Raízes do Xingu), Brasil Novo, PA, 04 Apr. 2017 | 210082 | L | *P. trinitatis* | -3.3228 | -52.2747 | ON145667 | ON146208 | – |
| PES, Lagoa Santa, MG, 15 Dec. 2020 | 210084 | L | *G. soricina* | -19.5606 | -43.9667 | ON145669 | NA | – |

**Table S3** GenBank accession numbers for *Rickettsia* terminals included in phylogenetic analyses of gltA (citrate synthase) gene, along information on their host and the phenotype it causes.

| **Terminal** | **GenBank** | **Reference** | **Host** | **Phenotype** |
| --- | --- | --- | --- | --- |
| Group Rhyzobius | | | | |
| *Rickettsia* from *Polydrusus tereticollis* | OZ032142 |  | Coleoptera: Curculionidae |  |
| *Rickettsia* from *Rhyzobius litura* | FJ666753 | Weinert et al 2009 | Coleoptera: Coccinellidae |  |
| Group Meloidae | | | | |
| *Rickettsia* from Meloidae | FJ666754 | Weinert et al 2009 | Coleoptera: Meloidae |  |
| Group Belli | | | | |
| *Rickettsia* from *Bemisia tabaci* | DQ077708 | Weinert et al 2009 | Hemiptera: Aleyrodidae |  |
| Rickettsia ALSK | KX254162 |  | *Xenopsylla minax* (Siphonaptera: Pulicidae) |  |
| *Rickettsia* from *Carica papaya* and *Empoasca papayae* | U76908 | Weinert et al 2009 | Hemiptera: Cicadellidae | plant pathogen |
| *Rickettsia* RDa420 | AF497584 |  | *Dermacentor auratus* (Ixodida: Ixodidae) from a bear |  |
| *Rickettsia* from Bombyliid bee fly | FJ666755 | Weinert et al 2009 | Diptera: Bombyliidae |  |
| *Rickettsia* from *Acyrthosiphon pisum* | FJ666756 | Weinert et al 2009 | Hemiptera: Aphididae |  |
| *Rickettsia* from Bombyliid bee fly | FJ666757 | Weinert et al 2009 | Diptera: Bombyliidae |  |
| *Rickettsia* from *Brachys tessellatus* | FJ666758 | Weinert et al 2009 | Coleoptera: Buprestidae | male-killing |
| *Rickettsia* from Chrysopid lacewing | FJ666759 | Weinert et al 2009 | Neuroptera: Chrysopidae |  |
| *Rickettsia bellii* | CP000849 | Weinert et al 2009 | *Dermacentor variabilis* (Ixodida: Ixodidae) | vertebrate pathogen |
| *Rickettsia bellii* | CP000087 | Weinert et al 2009 | *Dermacentor variabilis* (Ixodida: Ixodidae) | vertebrate pathogen |
| *Rickettsia bellii* | MG550957 |  | *Amblyomma rotundatum* (Ixodida: Ixodidae) | vertebrate pathogen |
| *Rickettsia* from Elaterid beetle | FJ666760 | Weinert et al 2009 | Coleoptera: Elateridae |  |
| *Rickettsia* from Noctuid moth | FJ666761 | Weinert et al 2009 | Lepidoptera: Noctuidae |  |
| *Rickettsia* endosymbiont | OZ032152_ |  | *Lasioglossum_villosulum* (Hymenoptera: Apidae) |  |
| Group Adalia | | | | |
| *Rickettsia* from *Subcoccinella vigintiquatuorpunctata* | FJ666762 | Weinert et al 2009 | Coleoptera: Coccinellidae | male-killing |
| *Rickettsia* from *Adalia bipunctata* | FJ666765 | Weinert et al 2009 | Coleoptera: Coccinellidae | male-killing |
| *Rickettsia* from *Adalia bipunctata* | FJ666764 | Weinert et al 2009 | Coleoptera: Coccinellidae | male-killing |
| *Rickettsia* from *Adalia bipunctata* | FJ666763 | Weinert et al 2009 | Coleoptera: Coccinellidae | male-killing |
| *Rickettsia* from *Adalia bipunctata* | U59712 | Roux et al 1997 | Coleoptera: Coccinellidae | male-killing |
| *Rickettsia* from *Halyzia* | FJ666766 | Weinert et al 2009 | Coleoptera: Coccinellidae |  |
| *Rickettsia* from *Calvia quatuordecimguttata* | FJ666767 | Weinert et al 2009 | Coleoptera: Coccinellidae |  |
| *Rickettsia* from *Adalia decempunctata* | FJ666768 | Weinert et al 2009 | Coleoptera: Coccinellidae | male-killing |
| Group Canadensis | | | | |
| *Rickettsia canadensis* | CP000409 | Weinert et al 2009 | Ixodida: Ixodidae | vertebrate pathogen |
| *Candidatus* Rickettsia tarasevichiae | AF503167 | Weinert et al 2009 | *Ixodes persulcatus* (Ixodida: Ixodidae) |  |
| Group Spotted Fever | | | | |
| *Rickettsia helvetica* | U59723 | Roux et al 1997 | *Ixodes ricinus* (Ixodida: Ixodidae) | vertebrate pathogen |
| *Rickettsia monacensis* | LN794217 |  |  |  |
| Uncultured *Rickettsia* sp. | MG906649 | Jacinavicius et al 2019 | *Herpetacarus* *hertigi*, *Quadraseta trapezoides,* *Trombewingia* *bakeri* (Trombiculoidae) |  |
| Uncultured *Rickettsia* sp. | MG906658 | Bassini-Silva et al 2023 | *Eutrombicula tinami* (Trombiculoidae) |  |
| Uncultured *Rickettsia* sp. | MG906657 | Bassini-Silva et al 2023 | *Blankaartia sinnamaryi, Eutrombicula tinami* (Trombiculoidae) |  |
| *Rickettsia montana* | U74756 | Roux et al 1997 | *Microtus* sp. (Rodentia: Cricetidae) | vertebrate pathogen |
| *Rickettsia massiliae* | CP000683 | Weinert et al 2009 | Cells in culture | vertebrate pathogen |
| *Rickettsia japonica* | U59724 | Roux et al 1997 | *Homo sapiens* (Primates: Hominidae) | vertebrate pathogen |
| *Rickettsia peacockii* | HF935076 | Anstead and Chilton 2013 | *Ixodes angustus* (Ixodida: Ixodidae) |  |
| *Rickettsia rickettsii* | CP000848 | Weinert et al 2009 | *Homo sapiens* (Primates: Hominidae) | vertebrate pathogen |
| *Rickettsia conorii* | OP382371 | Xue et al 2023 | *Haemaphysalis concinna* (Ixodida: Ixodidae) | vertebrate pathogen |
| *Rickettsia sibirica* | MW802694 | Han et al. 2025 | *Rhipicephalus turanicus* (Ixodida: Ixodidae) | vertebrate pathogen |
| *Rickettsia slovaca* | CP002428 | Fournier et al 2012 | *Dermacentor* sp. (Ixodida: Ixodidae) | vertebrate pathogen |
| *Rickettsia amblyommatis* | CP012420 | Yen et al 2021 | *Amblyomma cajennense* (Ixodida: Ixodidae) | vertebrate pathogen |
| *Rickettsia* sp. | MN581991 |  | *Rhipicephalus* sp. (Ixodida: Ixodidae) *on Nesokia indica* (Rodentia: Muridae) |  |
| Candidatus *Rickettsia wissemanii* | LT558852 | Tahir et al 2016 | *Ornithodoros hasei* (Ixodida: Argasidae) on *Noctilio albiventris* (Chiroptera: Noctilionidae) |  |
| *Rickettsia parkeri* Atlantic Rainforest | CP040325 | Londoño et al 2019 | *Amblyomma ovale* (Ixodida: Ixodidae) | vertebrate pathogen |
| Candidatus *Rickettsia andeanae* | GU169051 | Jiang et al 2005 | *Ixodes boliviensis* (Ixodida: Ixodidae) |  |
| *Rickettsia rhipicephali* | CP003342 |  | *Rhipicephalus sanguineus* (Ixodida: Ixodidae) |  |
| *Rickettsia heilongjiangensis* | AY285776 | Shpynov et al 2006 | *Haemaphysalis concinna* (Ixodida: Ixodidae) | vertebrate pathogen |
| *Rickettsia africae* | HM050288 |  | *Rhipicephalus evertsi* (Ixodida: Ixodidae) |  |
| Group Typhus | | | | |
| *Rickettsia typhi* | U59714 | Roux et al 1997 | *Homo sapiens* (Primates: Hominidae) | vertebrate pathogen |
| *Rickettsia prowazekii* | U59715 |  | *Homo sapiens* (Primates: Hominidae) | vertebrate pathogen |
| Group Transitional | | | | |
| *Rickettsia australis* | U59718 | Roux et al 1997 | *Homo sapiens* (Primates: Hominidae) | vertebrate pathogen |
| *Rickettsia akari* | CP000847 | Weinert et al 2009 | *Homo sapiens* (Primates: Hominidae) | vertebrate pathogen |
| *Rickettsia* *felis* | KP870108 |  | *Ctenocephalides felis*  (Siphonaptera: Pulicidae) | vertebrate pathogen |
| Candidatus *Rickettsia senegalensis* P127 | MK548197 |  | *Ctenocephalides felis*  (Siphonaptera: Pulicidae) | vertebrate pathogen |
| *Rickettsia asembonensis* | MK031661 |  | *Ctenocephalides felis*  (Siphonaptera: Pulicidae) | vertebrate pathogen |
| *Rickettsia felis* (Arica) | JX402763 | Abarca et al 2013 | *Rhipicephalus sanguineus* (Ixodida: Ixodidae) | vertebrate pathogen |
| *Rickettsia* sp. | MG783574 | Bassini-Silva et al 2018 | *Blankaartia sinnamaryi* (Trombiculoidae) |  |
| *Rickettsia aeschilimannii* | KP675964 |  | *Hyalomma aegyptium* (Ixodida: Ixodidae) | vertebrate pathogen |
| Uncultured *Rickettsia* | MF175748 | Bitencourth et al 2017 | *Amblyomma sculptum* (Ixodida: Ixodidae) |  |

**References**

Abarca K, López J, Acosta-Jamett G, Martínez-Valdebenito C (2013) *Rickettsia felis* in *Rhipicephalus sanguineus* from two distant Chilean cities. Vector Borne Zoonotic Dis 13(8):607-9 https://doi.org/10.1089/vbz.2012.1201

Anstead CA, Chilton NB (2013) A Novel Rickettsia Species Detected in Vole Ticks (*Ixodes angustus*) from Western Canada. Appl. Environ. Microbiol. 79(24): 7583 https://doi.org/10.1128/AEM.02286-13

Bassini-Silva R, Jacinavicius FC, Maturano R, Muñoz-Leal S, Ochoa R, Bauchan G, Labruna MB, Barros-Battesti DM (2018) *Blankaartia sinnamaryi* (Trombidiformes: Trombiculidae) parasitizing birds in southeastern Brazil, with notes on *Rickettsia* detection. Rev Bras Parasitol Vet. 27(3): 354-362. https://doi.org/10.1590/S1984-296120180057

Bitencourth K, Amorim M, DE Oliveira SV, Caetano RL, Voloch CM, Gazêta GS. (2017) *Amblyomma sculptum*: genetic diversity and rickettsias in the Brazilian Cerrado biome. Med Vet Entomol 31(4):427-437 https://doi.org/ 10.1111/mve.12249

Fournier PE, El Karkouri K, Robert C, Médigue C, Raoult D (2012) Complete genome sequence of *Rickettsia slovaca*, the agent of tick-borne lymphadenitis. J Bacteriol 194(6):1612 https://doi.org/10.1128/JB.06625-11

Han X, Liu Z, Jiang Z, Zhao S, Hornok S, Yang M, Liu G, Wang Y. (2025) Detection of spotted fever group rickettsiae and *Coxiella burnetii* in long-tailed ground squirrels (*Spermophilus undulatus*) and their ectoparasites. Front Vet Sci 12:1553152 https://doi.org/10.3389/fvets.2025.1553152

Jacinavicius FC, Bassini-Silva R, Muñoz-Leal S, Welbourn C, Ochoa R, Labruna MB, Barros-Battesti DM (2019) Molecular detection of *Rickettsia* genus in chigger mites (Trombidiformes: Trombiculidae) collected on small mammals in southeastern brazilian. Rev Bras Parasitol Vet 28(4): 563-568 https://doi.org/10.1590/S1984-29612019069

Jiang J, Blair PJ, Felices V, Moron C, Cespedes M, Anaya E, Schoeler GB, Sumner JW, Olson JG, Richards AL (2005) Phylogenetic analysis of a novel molecular isolate of spotted fever group *Rickettsiae* from northern Peru: Candidatus *Rickettsia andeanae*. Ann N Y Acad Sci 1063:337--342. https://doi.org/10.1196/annals.1355.054

Londoño AF, Mendell NL, Valbuena GA, Routh AL, Wood TG, Widen SG, Rodas JD, Walker DH, Bouyer DH (2019 ) Whole-Genome Sequence of *Rickettsia parkeri* Strain Atlantic Rainforest, Isolated from a Colombian Tick. Microbiol Resour Announc 8(39):e00684-19 https://doi.org/10.1128/MRA.00684-19

Macaluso KR, Sonenshine DE, Ceraul SM, Azad AF. (2002) Rickettsial infection in *Dermacentor variabilis* (Acari: Ixodidae) inhibits transovarial transmission of a second *Rickettsia*. J Med Entomol 39(6):809-813. https://doi.org/10.1603/0022-2585-39.6.809

Roux V, Rydkina E, Eremeeva M, Raoult D. (1997) Citrate synthase gene comparison, a new tool for phylogenetic analysis, and its application for the rickettsiae. Int J Syst Bacteriol 47(2):252-61. https://doi.org/10.1099/00207713-47-2-252

Shpynov SN, Fournier PE, Rudakov NV, Samoilenko IE, Reshetnikova TA, Yastrebov VK, Schaiman MS, Tarasevich IV, Raoult D (2006) Molecular identification of a collection of spotted Fever group rickettsiae obtained from patients and ticks from Russia. Am J Trop Med Hyg 74(3):440-3

Tahir D, Socolovschi C, Marié JL, Ganay G, Berenger JM, Bompar JM, Blanchet D, Cheuret M, Mediannikov O, Raoult D, Davoust B, Parola P (2016) New *Rickettsia* species in soft ticks *Ornithodoros hasei* collected from bats in French Guiana. Ticks Tick Borne Dis 7(6):1089-1096 https://doi.org/10.1016/j.ttbdis.2016.09.004

Weinert LA, Werren JH, Aebi A, Stone GN, Jiggins FM (2009) Evolution and diversity of *Rickettsia* bacteria. BMC Biol 7: 6

Xue J, Ren Q, Yang XL, Wang J, Xie G, Du L, Guo WP (2023) Human pathogens in ticks removed from humans in Hebei, China. Heliyon. 9(3):e13859 https://doi.org/10.1016/j.heliyon.2023.e13859

Yen WY, Stern K, Mishra S, Helminiak L, Sanchez-Vicente S, Kim HK (2021) Virulence potential of *Rickettsia amblyommatis* for spotted fever pathogenesis in mice. Pathog Dis 79(5):ftab024. https://doi.org/ 10.1093/femspd/ftab024

**Table S4** GenBank accession numbers for *Rickettsia* sequences employed in the multiloci analyses, grouped according to their phylogenetic or serological groups. Abbreviations: 16SrDNA, Small Subunit Bacterial Ribosomal DNA); gltA, citrate synthase; atpA, ATP synthase subunit alpha; and CoxA, bacterial cytochrome C oxidase subunit I.

| **Terminal** | **16SrDNA** | **gltA** | **AtpA** | **CoxA** | |
| --- | --- | --- | --- | --- | --- |
| Group Rhyzobius | | | | | |
| *Rhyzobius litura* | FJ609388 | FJ666753 | FJ666796 | FJ666774 | |
| Group Meloidae | | | | | |
| Meloidae | FJ609389 | FJ666754 | FJ666797 | FJ666775 | |
| Group Belli | | | | | |
| *Bemisia tabaci* | DQ077707 | DQ077708 | – | – | |
| *C. papaya* and *E. papayae* | U76910 | U76908 | – | – | |
| Bombyliid bee fly (strain WOL150801) | FJ609390 | FJ666755 | FJ666798 | FJ666776 | |
| *Acyrthosiphon pisum* | *FJ609391* | FJ666756 | FJ666799 | FJ666777 | |
| Bombyliid bee fly (strain WOL150653) | FJ609392 | FJ666757 | FJ666800 | FJ666778 | |
| *Brachys tessellatus* | FJ609393 | FJ666758 | FJ666801 | – | |
| Chrysopid lacewing | FJ609395 | FJ666759 | – | FJ666780 | |
| *Rickettsia bellii* (OSU 85-389) | CP000849 | CP000849 | CP000849 | CP000849 | |
| *Rickettsia bellii* (RML369-C) | CP000087 | CP000087 | CP000087 | CP000087 | |
| Elaterid beetle | FJ609396 | FJ666760 | FJ666802 | FJ666781 | |
| Noctuid moth | FJ609397 | FJ666761 | FJ666803 | FJ666782 | |
| Group Adalia | | | | | |
| *Subcoccinella vigintiquatuorpunctata* | FJ609398 | FJ666762 | FJ666804 | FJ666783 | |
| *Adalia bipunctata* (strain 10J) | FJ609400 | FJ666765 | FJ666807 | FJ666787 | |
| *Adalia bipunctata* (strain 9I) | FJ609401 | FJ666764 | FJ666808 | FJ666786 | |
| *Halyzia* | FJ609402 | FJ666766 | FJ666809 | FJ666788 | |
| *Calvia quatuordecimguttata* | FJ609403 | FJ666767 | FJ666810 | FJ666789 | |
| *Adalia decempunctata* | FJ609404 | FJ666768 | FJ666811 | FJ666790 | |
| Group Canadensis | | | | | |
| *Rickettsia canadensis* | CP000409 | CP000409 | CP000409 | CP000409 | |
| Candidatus *Rickettsia tarasevichiae* | AF503168 | AF503167 | – | – | |
| Group Spotted Fever | | | | | |
| *Rickettsia helvetica* | OY974080 | OY974080 | OY974080 | OY974080 | |
| *Rickettsia monacensis* | LN794217 | LN794217 | LN794217 | LN794217 | |
| *Rickettsia montanensis* | CP003340 | CP003340 | CP003340 | CP003340 | |
| *Rickettsia massiliae* | CP000683 | CP000683 | CP000683 | CP000683 | |
| *Rickettsia japonica* | L36213 | U59724 | DQ821776 | – | |
| *Rickettsia peacockii* | DQ062433 | DQ100162 | – | – | |
| *Rickettsia rickettsii* | CP000848 | CP000848 | CP000848 | CP000848 | |
| *Rickettsia conorii* | AE006914 | AE006914 | AE006914 | AE006914 | |
| *Rickettsia sibirica* | KY780085 | KY780027 | – | – | |
| *Rickettsia slovaca* | CP002428 | CP002428 | CP002428 | CP002428 | |
| *Rickettsia amblyommatis* | CP012420 | CP012420 | CP012420 | CP012420 | |
| Candidatus *Rickettsia wissemanii* | LT558851 | LT558852 | – | – | |
| *Rickettsia parkeri* Atlantic Rainforest | CP040325 | CP040325 | CP040325 | CP040325 | |
| *Rickettsia rhipicephali* | CP003342 | CP003342 | CP003342 | CP003342 | |
|  | *Rickettsia heilongjiangensis* | CP002912 | CP002912 | CP002912 | CP002912 |
|  | *Rickettsia africae* | NC012633 | NC012633 | NC012633 | NC012633 |
| Group Typhus | | | | | |
| *Rickettsia typhi* | AE017197 | AE017197 | AE017197 | – | |
| *Rickettsi prowazekii* | NC017560 | NC017560 | NC017560 | NC017560 | |
| Group  Transitional | | | | | |
| *Rickettsia australis* | U17644 | U59718 | DQ821777 | – | |
| *Rickettsia akari* | CP000847 | CP000847 | CP000847 | CP000847 | |
| *Aulogymnus trilineatus* | FJ609405 | FJ666769 | FJ666812 | FJ666792 | |
| *Aulogymnus balani* | FJ609406 | FJ666770 | FJ666813 | FJ666793 | |
| *Pediobius rotundatus* | FJ609407 | FJ666771 | FJ666814 | FJ666794 | |
| *Rickettsia* *felis* (URRWXCal2) | CP000053 | CP000053 | CP000053 | CP000053 | |
| Candidatus *Rickettsia senegalensis* | KF666476 | KF666472 | – | – | |
| *Rickettsia asembonensis* | NZCP116496 | NZCP116496 | NZCP116496 | NZCP116496 | |
| *Rickettsia felis* (NTU-Lb) | OM912382 | OM936910 | – | – | |
| *Rickettsia aeschilimannii* | KT318741 | KT318743 | – | – | |
| Newly sequenced | | | | | |
| *A. dubitatum* (UFMG AC 170244) | PX530379 | PX584451 | PX584473 | PX584480 | |
| *O. aff saraivai* (UFMG AC 170467) | – | PX584455 | PX584479 | – | |
| *O.* sp Ubajara (UFMG AC 171333) | – | PX584458 | – | PX584481 | |
| *O. fonsecai* (UFMG AC 171498) | – | PX584457 | PX584478 | – | |
| *O. fonsecai* (UFMG AC 172234) | – | PX584453 | PX584472 | – | |
| *O.* sp2 (UFMG AC 180276) | – | PX584452 | PX584474 | – | |
| *O. fonsecai* (UFMG AC 210291) | – | PX584454 | PX584477 | PX584482 | |
| *W. pachywhartoni* (UFMG AC 210340) | PX530378 | PX584466 | PX584476 | – | |
